# Genome Assembly and Winged Fruit Gene Regulation of Chinese Wingnut: Insights from Genomic and Transcriptomic Analyses

**DOI:** 10.1093/gpbjnl/qzae087

**Published:** 2024-12-12

**Authors:** Fangdong Geng, Xuedong Zhang, Jiayu Ma, Hengzhao Liu, Hang Ye, Fan Hao, Miaoqing Liu, Meng Dang, Huijuan Zhou, Mengdi Li, Peng Zhao

**Affiliations:** Key Laboratory of Resource Biology and Biotechnology in Western China, Ministry of Education, College of Life Sciences, Northwest University, Xi’an 710069, China; Provincial Key Laboratory of Biotechnology of Shaanxi Province, College of Life Sciences, Northwest University, Xi’an 710069, China; Key Laboratory of Resource Biology and Biotechnology in Western China, Ministry of Education, College of Life Sciences, Northwest University, Xi’an 710069, China; Provincial Key Laboratory of Biotechnology of Shaanxi Province, College of Life Sciences, Northwest University, Xi’an 710069, China; Key Laboratory of Resource Biology and Biotechnology in Western China, Ministry of Education, College of Life Sciences, Northwest University, Xi’an 710069, China; Key Laboratory of Resource Biology and Biotechnology in Western China, Ministry of Education, College of Life Sciences, Northwest University, Xi’an 710069, China; Key Laboratory of Resource Biology and Biotechnology in Western China, Ministry of Education, College of Life Sciences, Northwest University, Xi’an 710069, China; College of Forestry, Northwest A&F University, Yangling 712100, China; Key Laboratory of Resource Biology and Biotechnology in Western China, Ministry of Education, College of Life Sciences, Northwest University, Xi’an 710069, China; Key Laboratory of Resource Biology and Biotechnology in Western China, Ministry of Education, College of Life Sciences, Northwest University, Xi’an 710069, China; Xi’an Botanical Garden of Shaanxi Province, Institute of Botany of Shaanxi Province, Xi’an 710061, China; Key Laboratory of Resource Biology and Biotechnology in Western China, Ministry of Education, College of Life Sciences, Northwest University, Xi’an 710069, China; Key Laboratory of Resource Biology and Biotechnology in Western China, Ministry of Education, College of Life Sciences, Northwest University, Xi’an 710069, China

**Keywords:** Chinese wingnut, Genome, Starch and sucrose metabolism, Subgenome, Winged fruit

## Abstract

The genomic basis and biology of winged fruit are interesting issues in ecological and evolutionary biology. Chinese wingnut (*Pterocarya stenoptera*) is an important horticultural and economic tree species in China. The genomic resources of this hardwood tree could advance the genomic studies of Juglandaceae species and elucidate their evolutionary relationships. Here, we reported a high-quality reference genome of *P*. *stenoptera* (N50 = 35.15 Mb) and performed a comparative genomic analysis across Juglandaceae species. Paralogous relationships among the 16 chromosomes of *P*. *stenoptera* revealed eight main duplications representing the subgenomes. Molecular dating suggested that the most recent common ancestor of *P. stenoptera* and *Cyclocarya paliurus* diverged from *Juglans* species around 56.7 million years ago (MYA). The expanded and contracted gene families were associated with cutin, suberine, and wax biosynthesis, cytochrome P450, and anthocyanin biosynthesis. We identified large inversion blocks between *P*. *stenoptera* and its relatives, which were enriched with genes involved in lipid biosynthesis and metabolism, as well as starch and sucrose metabolism. Whole-genome resequencing of 28 individuals revealed clearly phylogenetic clustering into three groups corresponding to *Pterocarya macroptera*, *Pterocarya hupehensis*, and *P*. *stenoptera*. Morphological and transcriptomic analyses showed that *CAD*, *COMT*, *LOX*, and *MADS*-*box* play important roles during the five developmental stages of wingnuts. This study highlights the evolutionary history of the *P*. *stenoptera* genome and supports *P*. *stenoptera* as an appropriate Juglandaceae model for studying winged fruits. Our findings provide a theoretical basis for understanding the evolution, development, and diversity of winged fruits in woody plants.

## Introduction

The adaptation, reproduction, and evolution of flowering plants depend heavily on fruit traits, which are fundamental characteristics of angiosperms [1–3]. Fruits play an important role in plant reproduction efficiency, environmental adaptability, and species diversity [4–6]. Winged fruit may be the main reason for the rapid diffusion and differentiation of angiosperms in the early evolutionary stages. It is also a key innovative trait in angiosperms’ adaptation to wind-borne migration [2,7]. Winged fruit appears in at least 93 angiosperm families [8]; however, the evolution and development of winged fruit remain poorly understood, necessitating further investigation into the genetic mechanisms of winged fruit development and diversity.

Emergence, maintenance, and evolutionary fixation of innovative traits are an important basis for angiosperm speciation and diversification [9]. Winged fruit is a key trait in angiosperm fruits, allowing them to adapt to wind dissemination. The genus *Pterocarya*, belonging to Juglandaceae (Fagales), has important ecological, economic, and ornamental values [10–13], and comprise eight species mainly distributed in East Asia. This genus has three main types of wingnuts, which are morphologically distinct and representative: narrowed wings, elliptic–ovate wings, and elliptic–rhomboid wings [14]. Thus, *Pterocarya* provides good materials for studying the molecular basis of winged fruit evolution and diversity.

Recently, examples of high-quality woody tree genome research have been published on many species, including *Camellia sinensis* [15], *Torreya grandis* [16], *Malus* [17–19], *Quercus dentata* [20], *Metasequoia glyptostroboides* [21], *Rhoiptelea chiliantha* [22], *Cyclocarya paliurus* [23], and *Juglans nigra* [24]. These genomic studies have enabled researchers to identify ecological adaption genes and understand the underlying mechanisms of population genetics, agricultural traits, and important metabolic pathways in tree species. However, addressing the genetic basis of winged fruit development and the genetic variations in perennial species is challenging, mainly due to insufficient available reference genome resources. Therefore, a high-quality reference genome is an essential foundation for studying the development and evolution of winged fruit traits.

*P*. *stenoptera*, also known as Chinese wingnut, is a widely distributed and cultivated hardwood tree species in deciduous broad-leaved forests in China [12,25]. It is an important horticultural tree species widely used as economic plants. The bark and branches contain tannins and fibers, which are used as raw materials in various fields. Fruits can be used for feed and brewing, and seeds can be used for oil extraction. Previous studies have focused on the phylogeography [26], landscape genomics [27], and local adaptation [11] of *P*. *stenoptera*. Although a high-quality chromosome-level genome assembly is an essential genetic resource for understanding evolutionary history and fruit trait development in woody plants, genomic resources for *P*. *stenoptera* remain largely undeveloped. Recently, the scaffold-level genome of *P*. *stenoptera* has reported [28,29]; however, a high-quality reference genome is still required to verify transcription factor (TF) genes related to diverse phenotypes and development processes.

Here, we constructed a high-quality *de novo* chromosome-level genome assembly of Chinese wingnut by integrating Illumina HiSeq (short reads), PacBio (long reads), and high-throughput chromosome conformation capture (Hi-C) technologies. We identified Chinese wingnut-specific gene family expansions and analyzed variations in genome structure and population structure via whole-genome resequencing of 28 accessions from *Pterocarya macroptera*, *Pterocarya hupehensis*, and *P*. *stenoptera*. Furthermore, we characterized the evolution, size, and structure of the LOX gene family in *P*. *stenoptera*. To elucidate the molecular and metabolic mechanisms underlying winged fruit development, we also analyzed the morphological trails and transcriptomic profiles across five developmental stages [1 day after flowering (DAF), 15 DAF, 35 DAF, 45 DAF, and 75 DAF] of *P*. *stenoptera* wingnuts. The high-quality genome sequence of Chinese wingnut reported here has important implications for in-depth genomic studies of *P*. *stenoptera* and Juglandaceae species, providing insights into the genetic basis of winged fruit development and evolutionary diversity, as well as the genetic mechanisms of fruit innovation and seed dispersal by abiotic means.

## Results

### *De novo* assembly of a high-quality genome of *P*. *stenoptera*

**Figure 1:**
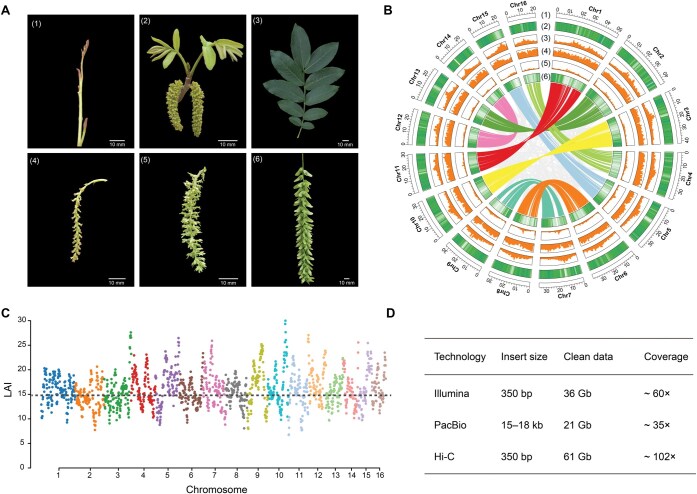
Landscape of the morphology and genome assembly of *P. stenoptera* **A.** Morphology of *P*. *stenoptera* for fresh branch and buds (1), leaves and male flower catkins (2), leaves (3), female flowers (4), mature female flowers (5), and winged fruits (6). **B.** Circos plot showing the genome features of *P*. *stenoptera*. (1) Number of chromosomes (the unit is Mb); (2) LTR/*Copia* retrotransposon density; (3) LTR/*Gypsy* retrotransposon density; (4) TE density; (5) GC content; and (6) gene density. **C.** LAI assessment for each assembled *P*. *stenoptera* chromosome. Dashed line (LAI = 15) indicates the average LAI value. **D.** Summary of sequencing data of *P*. *stenoptera* genome assembly. LTR, long terminal repeat; TE, transposable element; LAI, LTR Assembly Index; Chr, chromosome; Hi-C, high-throughput chromosome conformation capture.

We assembled the complete genome of Chinese wingnut (*P*. *stenoptera*) by combining Illumina HiSeq, PacBio, and Hi-C technologies (**Figure 1**A–D; Table S1). The final genome was 555.2 Mb in length with a scaffold N50 of 35.15 Mb (Table S2), comparable to the genome size estimated by *k*-mer analysis (Figure S1). The scaffolds were further anchored onto 16 pseudochromosomes, covering 97.52% of the assembled sequences (Figure S2; Table S3). The lengths of the 16 chromosomes of *P*. *stenoptera* ranged from 22,855,037 bp to 51,457,875 bp (Table S4). To assess the completeness and accuracy of the *P*. *stenoptera* genome assembly, we employed five analytical methods. First, 98.4% of the benchmarking universal single-copy orthologs (BUSCO) genes were verified in the genome (Table S5) [30]. Second, a total of 238 core eukaryotic genes (95.97%) were identified in the *P*. *stenoptera* genome assembly using the core eukaryotic genes mapping approach (CEGMA) [31]. Third, alignment of the clean Illumina short reads (36 Gb) to the assembled *P*. *stenoptera* genome yielded a mapping rate of 98.03%. Fourth, the quality value of genomic bases was 44.2 and accuracy was 99.99%. Fifth, the long terminal repeat (LTR) Assembly Index (LAI) of the *P*. *stenoptera* genome was ∼ 15 (Figure 1C), which indicates the gold-standard quality level of the assembly [32]. Moreover, comparison between our chromosome-level assembly and two previously reported scaffold-level *P*. *stenoptera* genomes [28,29] demonstrated the high-quality of our genome assembly (Figure S3; Tables S6 and S7).

A total of 29,820 protein-coding genes were predicted from the *P*. *stenoptera* genome assembly with an average coding sequence (CDS) length of 1176 bp (Table S6). Among them, 28,877 (96.8%) genes were annotated in the non-redundant (NR) database [33], 28,156 (94.4%) genes were annotated in InterPro [34], 22,943 (76.9%) genes were annotated in the Swiss-Prot protein sequence database [35], 22,663 (76.0%) genes were annotated in Kyoto Encyclopedia of Genes and Genomes (KEGG) [36], and 22,471 (75.36%) genes were annotated in the Pfam protein families database [37], and 17,543 (58.8%) genes were annotated in Gene Ontology (GO) (Table S8) [38]. A large number of non-coding RNAs were also annotated, including 8939 ribosomal RNAs (rRNAs), 902 transfer RNAs (tRNAs), 963 small nuclear RNAs (snRNAs), and 363 microRNAs (miRNAs) (Table S9). In addition, repetitive elements comprised 54.1% of the *P*. *stenoptera* genome. Specifically, LTR retrotransposons constituted 40.5% of the genome, with *Gypsy* and *Copia* elements accounting for 21.3% and 4.6% of the genome, respectively (Figure 1B).

### Population structure and demographic history of three *Pterocarya* species

**Figure 2:**
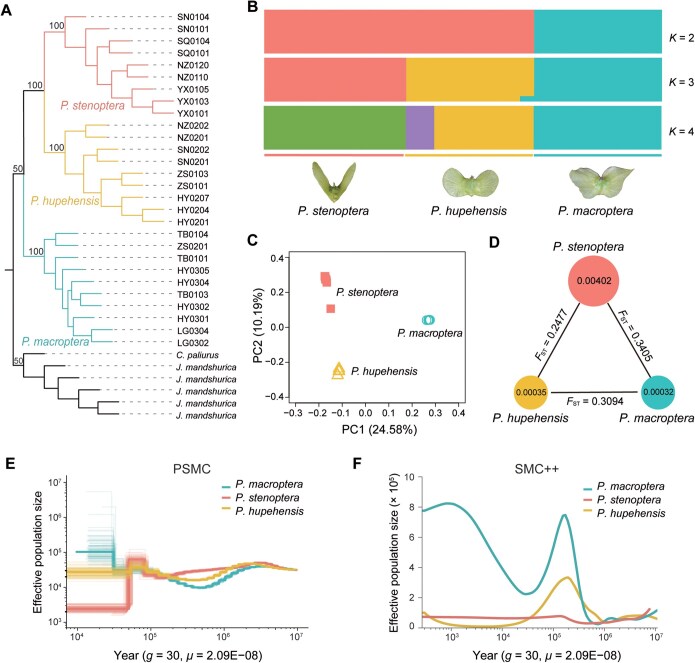
Population genomic analysis and demographic history of *P*. *stenoptera*, *P. macroptera*, and *P. hupehensis* **A**. The ML phylogenetic tree of 28 *Pterocarya* accessions. Individuals from *P*. *stenoptera*, *P. macroptera*, and *P. hupehensis* are represented by light red, light green, and yellow lines, respectively. **B**. Model-based population structure of 28 *Pterocarya* accessions (*K* = 2 to 4). **C**. PCA plot based on genetic covariance among all individuals of *P*. *stenoptera* (light red squares), *P*. *macroptera* (light green circles), and *P*. *hupehensis* (yellow triangles). **D**. Summary of nucleotide diversity (*θ*π) and population divergence (*F*_ST_) across three species. The size of each circle represents the nucleotide diversity (*θ*π) for the species, and values on the line between pairs indicate the population divergence (*F*_ST_). **E**. PSMC estimates of the effective population size changes for *P*. *stenoptera*, *P*. *macroptera*, and *P*. *hupehensis*. The time scale on the X-axis is calculated assuming neutral mutation rate per year (*μ*) = 2.09E−08 and generation time (*g*) = 30 years. **F**. SMC++ estimates of the effective population size changes for *P*. *stenoptera*, *P*. *macroptera*, and *P*. *hupehensis*. The time scale on the X-axis is calculated assuming neutral mutation rate per year (*μ*) = 2.09E−08 and generation time (*g*) = 30 years. ML, maximum likelihood; PCA, principal component analysis; PC, principal component; PSMC, pairwise sequentially Markovian coalescent; SMC++, sequential Markov coalescent++.

Whole-genome resequencing of 28 accessions (all individuals distributed in Qinling and Ta-pa Mountains, Shaanxi Province, China) resulted in 1404 Gb of clean Illumina short reads. All reads were mapped to the *P*. *stenoptera* reference genome (Table S10). Approximately 91.6%–99.4% of *P*. *stenoptera* sequence reads, 94.7%–99.4% of *P*. *hupehensis* sequence reads, and 87.6%–97.6% of *P*. *macroptera* sequence reads were accurately mapped to the reference genome, with mean depths of 37.3×, 37.6×, and 37.7×, respectively (Table S10). After quality control, a total of 38,120,880 high-quality single nucleotide polymorphisms (SNPs) from the 28 individuals were used to analyze the population genetic structure. Based on the phylogenetic tree, principal component analysis (PCA), and genetic structure analysis (where *K* = 3 is the optimal *K* value), these 28 trees were clustered into three groups corresponding to the three species: *P*. *macroptera*, *P*. *hupehensis*, and *P*. *stenoptera* (**Figure 2**A–C, Figure S4). The results of the three genetic groups were consistent with the morphological features (Figure 2). The nucleotide diversity (*θ*π) of *P*. *stenoptera* was the highest, while that of *P*. *macroptera* was lowest (Figure 2D). The genetic differentiation (*F*_ST_) between the *P*. *macroptera* and *P*. *stenoptera* was highest (*F*_ST_ = 0.3405), while that between *P*. *hupehensis* and *P*. *stenoptera* was lowest (*F*_ST_ = 0. 2477) (Figure 2D).

Both the pairwise sequentially Markovian coalescent (PSMC) and sequential Markov coalescent++ (SMC++) analyses [39,40] showed that the demographic histories of the three *Pterocarya* species were similar before ∼ 2 million years ago (MYA), which may represent the divergence time among those three species (Figure 2E and F). The effective population size declined at ∼ 1 MYA for the three *Pterocarya* species, and then increased and arrived at its peak between ∼ 0.5 MYA and 0.2 MYA except *P. stenoptera* (Figure 2E and F). Moreover, recent demographic histories based on SMC++ analysis revealed that the effective population size of *P*. *macroptera* experienced decline between ∼ 0.2 MYA and 0.03 MYA and then increased continuously, while that of *P*. *hupehensis* experienced decline between ∼ 0.2 MYA and 0.01 MYA. The effective population size of *P*. *stenoptera* stabilized after ∼ 0.2 MYA (Figure 2F).

### Whole-genome duplication and subgenomes of Chinese wingnut

**Figure 3:**
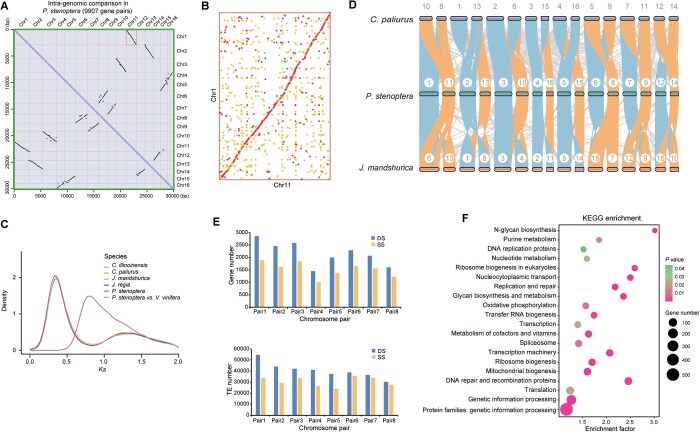
Common WGDs and subgenomes in *P*. *stenoptera* **A**. Dot-plot alignments between the chromosomes of the assembled *P*. *stenoptera* genome. The black dots represent homoeologous chromosomes within a genome. The dotted black syntenic lines indicate paralogues produced by the WGD event and γ whole-genome triplication. **B**. Dot-plot alignments between Chr1 and Chr11 of the assembled *P*. *stenoptera* genome. The red and yellow dots represent homologous regions with higher and lower similarity, respectively. **C**. Distribution of *Ks* values for syntenic genes in six species. Peaks indicate WGD events. **D**. Syntenic analysis among *P*. *stenoptera*, *C*. *paliurus*, and *J*. *mandshurica*. Orange lines indicate highly collinear homoeologous chromosomes across genomes, while blue lines indicate collinear inversions across genomes. The reference genomes are as follows: *C*. *paliurus* [23] and *J*. *mandshurica* [56]. **E**. Gene and TE counts in the subgenomes of the *P*. *stenoptera* assembly. **F**. KEGG enrichment analysis of genes in the DSs of the *P*. *stenoptera* assembly. WGD, whole-genome duplication; *Ks*, synonymous substitution rate; DS, dominant subgenome; SS, submissive subgenome; KEGG, Kyoto Encyclopedia of Genes and Genomes.

We investigated the whole-genome duplication (WGD) and its consequences in the Chinese wingnut (*P*. *stenoptera*) genome by comparing it to the genomes of four Juglandaceae species (*Juglans mandshurica*, *Carya illinoinensis*, *C*. *paliurus*, and *Juglans regia*) and the genome of *Vitis vinifera* (Figures S5 and S6). Paralogous relationships among the 16 chromosomes of the *P*. *stenoptera* genome revealed eight main duplications representing the subgenomes (Figure S7), jointly containing 9907 paralogous gene pairs in all collinear blocks of the *P*. *stenoptera* genome (**Figure 3**A, Figure S6). Both dot-plot alignments and paralogous block analyses in JCVI software showed eight main duplications within the assembled *P*. *stenoptera* chromosomes (Figure 3A and B, Figure S8). We observed similar WGD events using TBtools software (Figure S6) [41]. The synonymous substitution rate (*Ks*) peak occurred at ∼ 0.3 within the *P*. *stenoptera* assembly, demonstrating that *P*. *stenoptera* experienced one main WGD event (Figure 3C). Compared to the grape (*V*. *vinifera*) genome, *P*. *stenoptera* had one *Ks* peak at ∼ 0.75, which implied divergence between genes duplicated by γ whole-genome triplication (γWGT).

To confirm whether the *P*. *stenoptera* genome contains subgenomes, a synteny analysis was performed using TBtools software (Figure S7) [41]. Based on homology relationships, the genomes of three species, *P*. *stenoptera*, *C*. *paliurus*, and *J*. *mandshurica* were divided into two sets of homologous subgenomes. Thus, the 16 chromosomes in *P*. *stenoptera* were divided into eight chromosome pairs (Figure 3D). We further verified dominant subgenomes (DSs) and submissive subgenomes (SSs) according to the gene number, gene expression, and ancestral gene number in *P*. *stenoptera* (Figure 3E, Figure S9). Furthermore, the DSs contained more transposable elements (TEs) compared to SSs, the chromosome length of DSs was longer than that of SSs, and the ancestral gene number of DSs was more than that of SSs in *P*. *stenoptera* genome (Figure 3E, Figure S9; Table S11). The KEGG-enriched entries for genes specific to DSs and SSs were mostly the same, indicating that these specific genes may be functionally conserved in similar biological processes. In addition, DSs were enriched with genes related to transcription and translation, while SSs were enriched with genes related to resection and repair (Figure 3F, Figure S10).

### Phylogeny and gene family evolution of Chinese wingnut

**Figure 4:**
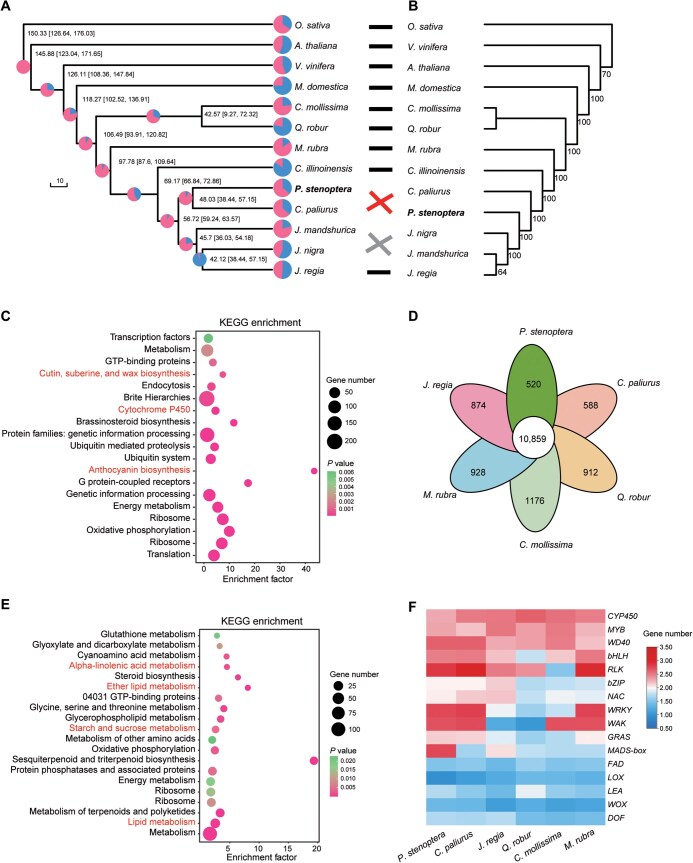
Chinese wingnut (*P*. *stenoptera*) genome evolution **A**. Expanded and contracted gene families of 13 species. The pie chart on each branch of the phylogenetic tree shows the proportions of expanded (pink) and contracted (blue) gene families, and the number near the node indicates the divergence time (MYA) with the numbers in parenthesis representing the 95% confidence interval. **B**. Phylogenetic tree of 13 species based on whole chloroplast genome data. The numbers near the nodes show the bootstrap values. **C**. KEGG enrichment analysis of the expanded and contracted gene families in the *P*. *stenoptera* assembly. **D**. Venn diagram showing the intersection of protein-coding genes among the six woody perennial species in Fagales. **E**. KEGG enrichment analysis of the 520 unique genes in *P*. *stenoptera* shown in (D). **F**. Heatmap showing the numbers of the *CYP450*, *MYB*, *WD40*, *bHLH*, *RLK*, *bZIP*, *NAC*, *WRKY*, *WAK*, *GRAS*, *MADS-box*, *FAD*, *LOX*, *LEA*, *WOX*, and *DOF* genes among *P*. *stenoptera*, *C*. *paliurus*, *J*. *regia*, *Q*. *robur*, *C*. *mollissima*, and *M*. *rubra*. MYA, million years ago.

To understand how the Chinese wingnut (*P*. *stenoptera*) genome evolved, we compared 13 plant genomes, including 6 species from the family Juglandaceae (**Figure 4**A; Table S12). A total of 30,903 gene families encompassing 47,4821 genes were identified and 174 species-specific gene families were obtained from *P*. *stenoptera*. Among the 13 species, 78 single-copy orthologous genes were identified and used to reconstruct the phylogenetic relationships of the 13 species (Figure 4A). The results of the phylogenetic tree showed that *P*. *stenoptera* and *C*. *paliurus* formed one clade, which was a sister group to *Juglans* (*J*. *mandshurica*, *J*. *nigra*, and *J*. *regia*). The fruits of the genera *Pterocarya* and *Cyclocarya* are winged fruits, while the other genera in the family Juglandaceae produce drupaceous nuts, consistent with the phylogenetic tree, indicating the closest genetic relationship between *P*. *stenoptera* and *C*. *paliurus* (Figure 4A). Molecular dating suggests that the most recently common ancestor of *P*. *stenoptera* and *C*. *paliurus* diverged from the genus *Juglans* around 56.7 MYA followed by the divergence of *P*. *stenoptera* and *C*. *paliurus* around 48.0 MYA. We also reconstructed their phylogenetic relationships based on CDSs of the chloroplast genome. The results showed that *P*. *stenoptera* and *Juglans* (*J*. *mandshurica*, *J*. *nigra*, and *J*. *regia*) formed one clade, creating a sister group to *C*. *paliurus* (Figure 4B). The inconsistency between the phylogenetic trees constructed based on chloroplast and nuclear genes suggests that the genus *Pterocarya* may have experienced ancient hybridization or gene introgression between *Cyclocarya* and *Juglans.* The same results were obtained in a previous study about the phylogeny of Juglandeae [42]. The phylogenetic positions of *J*. *regia* were also inconsistent between the phylogenetic trees constructed based on chloroplast and nuclear genes, suggesting that *J*. *regia* may be a product of ancient hybridization. A previous study on *J*. *regia* also supported this hypothesis [43]. To further explain the discordant phylogenetic signals, MSCquartets analysis [44] was performed for *P. stenoptera* and *J*. *regia*, and the results supported the presence of ancient hybridization or gene flow among species (Figure S11).

Compared to other plant species, a total of 803 gene families were expanded, and 1424 gene families were contracted in the *P*. *stenoptera* genome (Figure 4A; Table S12). The expanded and contracted gene families were associated with cutin, suberine, and wax biosynthesis, cytochrome P450, and anthocyanin biosynthesis (Figure 4C). We compared the protein-coding genes among six Fagales species (*P*. *stenoptera*, *J*. *regia*, *C*. *paliurus*, *Castanea mollissima*, *Quercus robur*, and *Myrica rubra*), and found that 520 specific genes were identified in *P*. *stenoptera*, which were associated with alpha-linolenic acid metabolism, starch and sucrose metabolism, and lipid metabolism (Figure 4D and E). We then analyzed the numbers of *CYP450*, *MYB*, *WD40*, *bHLH*, *RLK*, *bZIP*, *NAC*, *WRKY*, *WAK*, *GRAS*, *MADS-box*, *FAD*, *LOX*, *LEA*, *WOX*, and *DOF* genes across these six genomes, and found that the number of *MADS-box* genes was markedly higher in *P*. *stenoptera* than that in the other five genomes (Figure 4F).

### Genome-wide variations between *P*. *stenoptera*, *C*. *paliurus*, and *J*. *mandshurica* of the family Juglandaceae

**Figure 5:**
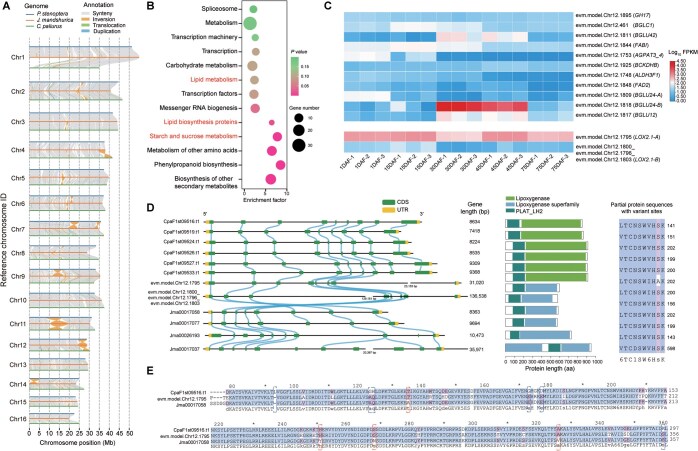
Comparative genomic analyses among *P*. *stenoptera*, *C*. *paliurus*, and *J*. *mandshurica* **A**. Genome collinearity among three Juglandaceae assemblies (*P*. *stenoptera*, *J*. *mandshurica*, and *C*. *paliurus*). For details of the Juglandaceae assemblies see Table S12. **B**. KEGG enrichment analysis of genes within the inversion region on Chr12 between *P*. *stenoptera* and *J*. *mandshurica* shown in (A). **C**. Expression heatmap of genes enriched in lipid metabolism during five developmental stages (1 DAF, 15 DAF, 35 DAF, 45 DAF, and 75 DAF) of *P*. *stenoptera* winged fruits. **D**. Gene structure, synteny, conserved domains, and partial protein sequences of six *LOX* genes from *C*. *paliurus* (CpaF1st09516.t1, CpaF1st09519.t1, CpaF1st09524.t1, CpaF1st09526.t1, CpaF1st09527.t1 9309, and CpaF1st09533.t1), two *LOX* genes from *P*. *stenoptera* (evm.model.Chr12.1795 and evm.model.Chr12.1780_evm.model.Chr12.1796_evm.model.Chr12.1803), and four *LOX* genes from *J*. *mandshurica* (Jma00017058, Jma00017077, Jma00026193, and Jma00017037) located within the inversion region on Chr12 shown in (A). The right panel shows a comparison of variant sites in randomly selected partial protein sequences between the Chr12 assemblies from three species. **E**. Protein sequence alignment of three *LOX* genes (CpaF1st09516.t1, evm.model.Chr12.1795, and Jma00017058) from *C*. *paliurus*, *P*. *stenoptera*, and *J*. *mandshurica*, respectively. The white/purple backgrounds highlight variant sites. Blue boxes indicate amino acid variations in *C*. *paliurus* compared to *P*. *stenoptera* and *J*. *mandshurica*, while red boxes indicate amino acid variations in *J*. *mandshurica* compared to *P*. *stenoptera* and *C*. *paliurus*. DAF, days after flowering; FPKM, fragments per kilobase of exon model per million mapped fragments; CDS, coding sequence; UTR, untranslated region.

We performed chromosome-level comparisons between the genomes of *P*. s*tenoptera*, *C*. *paliurus*, and *J*. *mandshurica* belonging to the family Juglandaceae (**Figure 5**A). We identified large syntenic blocks between *P*. *stenoptera* and its relatives *C*. *paliurus* and *J*. *mandshurica* (Figure 5A). There were large inversions between *P*. *stenoptera and J*. *mandshurica* on chromosome 7 (Chr7), Chr9, Chr11, Chr12, and Chr14, as well as between *C*. *paliurus* and *J*. *mandshurica* on Chr4, Chr11, and Chr12 (Figure 5A). KEGG enrichment analysis of the genes in the inversion region on Chr12 between *P*. *stenoptera* and *J*. *mandshurica* showed that these genes were mostly enriched in lipid metabolism, lipid biosynthesis proteins, and starch and sucrose metabolism (Figure 5B). The differences in synteny were anticipated to contribute to differences in the evolution of chromosomes among the three Juglandaceae species (Figure 5A).

To reveal the evolution and profile the transcriptome of genes associated with the development of *P*. *stenoptera* fruits, the expression levels of genes enriched in lipid metabolism, lipid biosynthesis proteins, and starch and sucrose metabolism were tested in 15 samples collected from five developmental stages of winged fruits. A total of 13 differentially expressed genes (DEGs) were identified between samples at five collection time points by pairwise comparisons, under the threshold of |log_2_ fold change| ≥ 1 and adjusted *P* ≤ 0.05 (Figure 5C; Table S13). These include two *LOX* genes closely related to fruit development and metabolism, lipoxygenase 2-1 (*LOX2.1-A*; evm.model.Chr12.1795) and lipoxygenase 2-1 (*LOX2.1-B*; evm.model.Chr12.1800_evm.model.Chr12.1796_evm.model.Chr12.1803) [45,46]. Furthermore, we performed a genome-wide analysis of the lipoxygenase (LOX) gene family in *P*. *stenoptera*, *C*. *paliurus*, and *J*. *mandshurica*, by screening the lipoxygenase and PLAT/LH2 conserved domains. We identified 8, 12, and 10 *LOX* genes in *P*. *stenoptera*, *C*. *paliurus*, and *J*. *mandshurica*, respectively (Figure S12). Interestingly, six *LOX* genes from *C*. *paliurus* (CpaF1st09516.t1, CpaF1st09519.t1, CpaF1st09524.t1, CpaF1st09526.t1, CpaF1st09527.t19309, and CpaF1st09533.t1), two *LOX* genes from *P*. *stenoptera* (evm.model.Chr12.1795 and evm.model.Chr12.1800_evm.model.Chr12.1796_evm.model.Chr12.1803), and four *LOX* genes from *J*. *mandshurica* (Jma00017058, Jma00017077, Jma00026193, and Jma00017037) were located within the inversion region on Chr12 (Figure 5D), which showed high homology among the three species, especially for the six *LOX* genes in *C*. *paliurus*. However, these *LOX* genes exhibited varying lengths among their gene sequences, specifically for evm.model.Chr12.1800_evm.model.Chr12.1796_evm.model.Chr12.1803, evm.model.Chr12.1795, and Jma00017037, which contained huge intron regions of 129,181 bp, 23,153 bp, and 20,937 bp, respectively (Figure 5D). We selected three *LOX* genes, each from one species, which showed the highest homology between the three species, and performed protein sequence alignment analysis. There were a total of 18 amino acid variations in *P*. *stenoptera* compared to *C*. *paliurus* and *J*. *mandshurica*, 6 amino acid variations in *C*. *paliurus* compared to *P*. *stenoptera* and *J*. *mandshurica*, and 4 amino acid variations in *J*. *mandshurica* compared to *P*. *stenoptera* and *C*. *paliurus* (Figure 5E). The nonsynonymous/synonymous substitution rate ratios (*Ka*/*Ks*) between the three genes were smaller than 1, indicating that these three genes underwent purifying selection and may have evolved relatively slowly (Table S14). The evm.model.Chr12.1795 was highly expressed in *P*. *stenoptera* during all five developmental stages of fruits, especially at 15 DAF and 45 DAF. However, Jma000017058 displayed low expression during all five developmental stages of *J*. *mandshurica* fruits, especially at 15 DAF and 75 DAF, where it was almost not expressed. Quantitative real-time polymerase chain reaction (qRT-PCR) results showed the same trend as the transcriptomic results, which further proved the reliability of the results (Figure S13). Our results suggest that these protein sequence differences might contribute to differences in fruit development and evolution among the three Juglandaceae species (Figure 5E).

### Morphological features and gene expression patterns during winged fruit development

**Figure 6:**
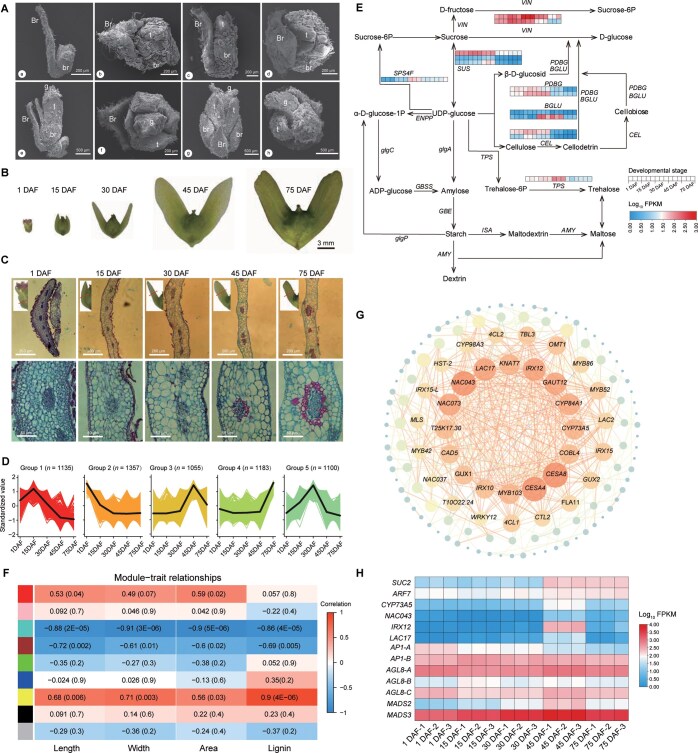
Dynamics of morphology development and gene expression in five developmental stages of *P*. *stenoptera* winged fruits **A**. SEM showing the developmental process of the *P*. *stenoptera* female flowers at four stages: Stage 1 (a and b), Stage 2 (c and d), Stage 3 (e and f), and Stage 4 (g and h). Images in (a, c, e, and g) are lateral view, while images in (b, d, f, and h) are vertical view. Br, large bract; br, bracteole; t, tepal; g, stigma. **B**. The floral morphology of *P*. *stenoptera* winged fruits at five developmental stages (1 DAF, 15 DAF, 30 DAF, 45 DAF, and 75 DAF). **C**. Paraffin sections of *P*. *stenoptera* fruit wings at five developmental stages (1 DAF, 15 DAF, 30 DAF, 45 DAF, and 75 DAF). The red lines in the upper panels indicate the crosscut positions. **D**. Classification of DEGs based on their expression patterns during five developmental stages using *K*-means analysis. The number in the parenthesis indicate the total DEG number in each group. **E**. Expression patterns of genes in the starch and sucrose pathway at five developmental stages. **F**. WGCNA showing the relationships between gene modules and four phenotypic traits of fruit wings (length, width, area, and lignin) across the five developmental stages. **G**. Coexpression correlation map of genes with correlation coefficients greater than 0.85. The size of each node in the map indicates its connectivity. **H**. Heatmap showing the expression patterns of *SUC2*, *ARF7*, lignin synthesis-related genes, and *MADS-box* genes during five developmental stages of *P*. *stenoptera* winged fruits. SEM, scanning electron microscopy; DEG, differentially expressed gene; WGCNA, weighted gene coexpression network analysis.

To understand the transition of winged fruits from flower buds to mature fruits, we first traced the developmental changes in female flowers and fruit morphology (**Figure 6**A and B). At developmental stage 1 [21 days before flowering (DBF)], the female flower tissue is enveloped by a large bract and two small bracts (*i.e.*, bracteoles). Then, the tepal extends its bracts, and the bracteoles also elongate at stage 2 (14 DBF). At stage 3 (7 DBF), the stigma protrudes from the tepal and becomes bifid. At stage 4 (1 DBF), the female flower and two bracteoles undergo further development, and the length of the bracteoles is greater than that of the large bract (Figure 6A). Subsequent observation of fruit development revealed that the large bract stops developing during this process, while the two bracteoles continue to develop, ultimately forming two wings of the fruit (Figure 6B). Paraffin section staining of fruit wings showed that the number of cell layers was significantly reduced while the cell volume was significantly increased in wings during the early developmental stages (from 1 DAF to 30 DAF). Moreover, the peripheral cells of the vascular bundle in wings were significantly red from 30 DAF to 75 DAF, indicating that the cell wall is lignified during the late developmental stages (Figure 6C).

To reveal transcriptomic patterns related to fruit wing development, we constructed 15 RNA sequencing (RNA-seq) libraries at five developmental stages of winged fruits (Figure S14; Table S15). We obtained 42.56 Gb of data with average 43,858,996 clean reads per library. To infer candidate genes associated with fruit wing development, a total of 5830 DEGs were identified by comparative transcriptomic analysis. All DEGs were categorized into five groups based on their expression patterns by *K*-means analysis (Figure 6D). DEGs in Group 1 and Group 5 displayed highest expression at 15 DAF and 30 DAF, respectively, corresponding to the early developmental stages of fruit wings. Among these genes, 34 DEGs were significantly enriched in pathways associated with starch and sucrose metabolism and fruit development [including *CEL* (evm.model.Chr2.855 and evm.model.Chr13.395), *SPS4F* (evm.model.Chr3.2972), *PDBG* (evm.model.Chr9.736 and evm.model.Chr7.1589), *BGLU40* (evm.model.Chr13.780), *BGLU* (evm.model.Chr12.1811 and evm.model.Chr12.1818), *VIN* (evm.model.3.2209 and evm.model.Chr5.1923), *SUS* (evm.model.Chr10.720, evm.model.Chr3.2361, and evm.model.Chr16.123), and *TPS1* (evm.model.Chr7.1230)] (Figure 6E, Figure S15 A and B; Table S16). DEGs in Group 3 exhibited highest expression at 45 DAF, corresponding to the transition process of fruit wing lignification. These DEGs were enriched in the phenylpropanoid biosynthesis and phenylalanine metabolic pathways (Figure S15C). To elucidate regulatory gene networks during fruit wing development, we performed weighted gene coexpression network analysis (WGCNA) to construct coexpression networks. The associations between gene modules and four phenotypic traits of fruit wings (length, width, area, and lignin) were analyzed across the five developmental stages via WGCNA (Figure 6F; Table S17). This analysis identified nine distinct gene modules, with the yellow module most strongly correlated with fruit wing lignification. By intersecting the DEGs in Group 3 with the yellow gene module, 371 genes were identified to be associated with fruit wing lignification. Protein interaction analysis further revealed key candidate genes involved in lignin synthesis, including *CYP73A5* (evm.model.Chr12.616), *NAC043* (evm.model.Chr11.1774), *IRX12* (evm.model.Chr7.222), and *LAC17* (evm.model.Chr7.357) (Figure 6G). In addition, considering that MADS-box TFs are closely associated with fruit development [47,48], we analyzed the MADS-box gene family in the *P*. *stenoptera* genome and identified 57 *MADS-box* TF genes. Among these, 4 TF genes were highly expressed across the five stages of winged fruit development (Figure 6H), including the agamous-like MADS-box protein AP1 gene *AP1-B* (evm.model.Chr13.746), the agamous-like MADS-box protein AGL8 homolog genes [*AGL8-A* (evm.model.Chr5.631) and *AGL8-C* (evm.model.Chr15.1485)], and the agamous-like MADS-box protein gene *MADS3* (evm.model.Chr3.2152). Moreover, the *ARF7*–*SUC2* module, which has been reported to play a role in the persistence of reproductive organs [49], was found to be consistently and highly expressed at the two stages (45 DAF and 75 DAF) of winged fruit development (Figure 6H; Table S18).

## Discussion

*P*. *stenoptera* (Chinese wingnut) is widely distributed and cultivated in China as a valuable economic tree for landscaping and ornamental purposes [10,12,14,25,50]. High-quality genomes play an important role in evolutionary and genetic studies. Although the scaffold-level *P*. *stenoptera* genome assembles using the short-read sequencing platform Illumina have been reported [28,29], a chromosome-level reference genome assembly of *P*. *stenoptera* will provide valuable biological genetic information for the in-depth study of population genetics and winged fruit development. In this study, we assembled a high-quality chromosome-level genome of *P*. *stenoptera* by combining Illumina HiSeq (short reads), PacBio (long reads), and Hi-C technologies (Figure 1). The scaffold N50 size was 35,148,204 bp, much higher than the previously reported *P*. *stenoptera* genomes [28,29]. The whole genome size was 555,202,549 bp, comparable to the previous genome versions. The chromosome-level reference genome of *P*. *stenoptera* was anchored into 16 pseudochromosomes with 29,820 protein-coding genes (Figure 1; Table S6). We identified a WGD event in *P*. *stenoptera* that occurred after it diverged from the common ancestor of the family Juglandaceae. This WGD may be related to the evolution of the subgenome in this ancient woody plant (Figure 3). We found that the DSs contained more TEs and genes as well as longer chromosomes than the SSs of *P*. *stenoptera* (Figure 3, Figure S9). There were many common events in angiosperms (including ancient polyploidization) that acted as an important evolutionary force for driving divergence and speciation [51,52].

Woody trees have adapted to various environmental conditions during their long evolutionary history. We found that 28 individuals of three *Pterocarya* species (*P*. *macroptera*, *P*. *hupehensis*, and *P*. *stenoptera*) were clearly divided into three groups based on phylogenetic trees, PCA, and structural analysis (Figure 2A–C). Although the distributions of these 28 individuals showed some overlap in Shaanxi Province in China, genetic differentiation (*F*_ST_) and morphological differences between the three *Pterocarya* species were relatively high (Figure 2). Their similar demographic histories until ∼ 2 MYA suggest a possible divergence time between these three *Pterocarya* species (Figure 2E and F).

Winged fruit is an important fruit type, which typically adapts to the wind for the dispersal of its seeds. In the family Juglandaceae, plants have both winged and wingless fruit types [53]. For instance, walnut (*Juglans*) and pecan (*Carya*) have wingless fruits, while *Platycarya*, *Cyclocarya*, and *Pterocarya* have winged fruits. Nonetheless, winged fruit is accepted as an ancestral character of Juglandaceae [52,54,55]. Previous studies have shown that the genera with winged and wingless fruits have diverged or diversified, likely reflecting adaptations to changes in seed dispersal vectors [2,5,7]. Comparative genomic analyses of 13 plant species revealed that the phylogenetic positions of *P*. *stenoptera* and *C*. *paliurus* conflicted between analyses based on nuclear data and chloroplast data (Figure 4), indicating that the species of the genera *Cyclocarya*, *Pterocarya*, and *Juglans* may have experienced ancient hybridization and gene introgression events [23,56,57]. The expanded and contracted gene families in *P*. *stenoptera* were mainly involved in cutin, suberine, and wax biosynthesis, cytochrome P450, and anthocyanin biosynthesis, and the specific genes in *P*. *stenoptera* compared to other five Fagales species (*J*. *regia*, *C*. *paliurus*, *C. mollissima*, *Q. robur*, and *M. rubra*) were associated with alpha-linolenic acid metabolism, ether lipid metabolism, and starch and sucrose metabolism (Figure 4). Those genes are responsible for the characterization and evolution of *P*. *stenoptera*. We identified the *LOX* genes in *P*. *stenoptera* (winged fruit), *C*. *paliurus* (winged fruit), and *J*. *mandshurica* (wingless fruit), which showed different gene clusters and structures as well as protein sequence variations between these three species (Figure 5).

Previous studies have elucidated the gene regulatory patterns underlying fruit development, morphology, and diversity [58–60]. However, the genetic basis of the evolution diversity and development of winged fruits is largely unknown. Comparative transcriptomic analysis of *P*. *stenoptera* at five developmental stages of winged fruits revealed 5830 DEGs, which were associated with lipid biosynthesis and metabolism, environmental adaptation, and starch and sucrose metabolism (Figure 6, Figure S15). We investigated the molecular basis and gene expression patterns underlying *P*. *stenoptera* winged fruit development by combining anatomical, genomic, and transcriptomic analyses. We then evaluated all candidate genes involved in stilbene synthesis in *P*. *stenoptera.* The essential genes responsible for lignin biosynthesis, lipid metabolism, and starch and sucrose metabolism were multiple-copy genes. We identified three *SUS* genes, two *BGLU* genes, two *VIN* genes, two *CEL* genes, two *PDBG* genes, one *TPS* gene, one *TPS* gene, two *SPS4F* genes, one *CYP73A5* gene, one *NAC043* gene, one *IRX12* gene, and one *IAC17* gene, which were DEGs in five fruit wing developmental stages (Figure 6E–F; Tables S16 and S18). Four *MADS-box* genes are continuously expressed during the development of the winged fruits. The fruit wing is an accessory organ of the fruit, derived from the bract. The first step in fruit wing development from the bract is to maintain persistence, followed by cell proliferation and growth. Organ shedding often occurs due to a lack of nutrients and competition for carbohydrates [61]. Sucrose can serve as the main carbohydrate/energy source for over long-distance transport. In this process, the *ARF7*–*SUC2* module participates in the inhibition of organ shedding by inducing sucrose transport in response to auxin [49]. *VIN* can break down sucrose into glucose and fructose, which can double the osmotic effect. It also mediates the sugar signaling pathway and regulates the expression of genes related to cell cycle, cell division, cell proliferation, and growth hormone synthesis [62,63]. *SUS* can catalyze the reversible conversion of sucrose into uridine diphosphate glucose (UDP-glucose) and fructose. It regulates the biological synthesis of cellulose, starch, lipids, and proteins during fruit development [64]. *BGLU* is involved in important physiological processes such as cell wall lignification, enzyme activity regulation, signal transduction, hormone activation, and secondary metabolism in plants. It is also closely related to fruit development [65,66]. On the other hand, the lignification of the cell wall can make the fruit wing tougher, which is conducive to seed dispersal. We identified four key candidate genes involved in lignification.

In summary, our study highlights the evolutionary history of the *P*. *stenoptera* genome and supports the use of *P*. *stenoptera* as an appropriate Juglandaceae model for studying winged fruits. Our findings provide a theoretical basis for investigating the evolution, development, and diversity of winged fruits in woody plants, as well as help elucidate the genetic mechanisms of innovative fruit morphological traits.

## Materials and methods

### Sample collection, library construction, and sequencing

We collected healthy leaves from an adult tree of *P*. *stenoptera* (genotype SNHY001), growing in the Taiping National Forest Park, Huyi, Shaanxi Province, China (altitude: 530 m; 33.996°N, 108.713°E). The whole genomic DNA of SNHY001 was prepared from the young leaf samples using the Nanobind PanDNA kit (Catalog No. 103-260-300, PacBio, Menlo Park, CA). For short-read sequencing, a 150 bp paired-end DNA library was constructed and sequenced on the Illumina NovaSeq 6000 platform (Illumina, San Diego, CA). For PacBio long-read sequencing, a long-read DNA library with an average inset size of 20 kb was prepared and sequenced on the PacBio Sequel II platform (Novogene, Beijing, China). For Hi-C sequencing, a Hi-C library was prepared and sequenced on the Illumina NovaSeq 6000 platform (Illumina). We obtained ∼ 36 Gb (60×) of Illumina short reads, ∼ 21 Gb (35×) of PacBio long reads, and ∼ 61 Gb (102×) of Hi-C reads (Figure 1D) from raw data.

### Genome assembly and quality assessment

The Illumina raw short reads were assessed with SOAPdenovo2 software [67]. The genome size was estimated via 17-*k*-mer analysis (Figure S1) [68]. *De novo* assembly of *P*. *stenoptera* was generated using the Illumina short-read and PacBio long-read sequencing data with hifiasm software [69]. Based on the clean Hi-C data, scaffolds were anchored to 16 chromosomes using ALLHiC software [70], resulting in a scaffold N50 of ∼ 35.6 Mb (Table S2). The interaction heatmap of the 16 chromosomes of *P*. *stenoptera* was generated using HiC-Pro software (Figure S2) [71]. The genome annotation statistics were calculated using agat_sp_statistics.pl in AGAT software [72]. The completeness and accuracy of the *P*. *stenoptera* genome assembly were evaluated using five methods, including BUSCO [30], CEGMA [31], sequence consistency evaluation by BWA v0.7.17 [73], sequence accuracy evaluation by Merqury [74], and assembly continuity evaluation by LAI scores [32].

### Genome annotation

Gene structure annotation of the assembled genome was performed by combining transcriptome-assisted prediction, homology-based prediction, and *ab initio* prediction. For transcriptome-assisted prediction, RNA-seq data from 13 tissues (Table S19) were aligned to the reference genome using Hisat v2.0.4 [75] and StringTie v1.3.3 [76]. For homology-based prediction, protein sequences from *J*. *regia*, *J*. *mandshurica*, *C*. *mollissima*, *Q*. *lobata*, and *Arabidopsis thaliana* were utilized for prediction using tblastn v2.2.26 [77] and GeneWise v2.4.1 [78]. For *ab initio* prediction, AUGUSTUS [79], geneid v1.4 [80], GlimmerHMM v3.04 [81], and screening for non-acceptable polymorphisms (SNAP) [82] were used to predict protein-coding genes based on intrinsic genomic features. Then, EVidenceModeler [83] was utilized to integrate the gene sets predicted by the aforementioned methods into a non-redundant and comprehensive gene set. The functional annotation of the final gene set was evaluated using six databases, including NR [33], InterPro [34], Swiss-Prot [35], KEGG [36], Pfam [37], and Gene Ontology (GO) [38].

Repetitive element annotation was performed using RepeatMasker software [84] based on Repbase (http://www.girinst.org/repbase) database, complemented by tandem repeat detection through *ab initio* prediction using Tandem Repeats Finder (TRF) (http://tandem.bu.edu/trf/trf.html). Additionally, Repbase and a *de novo* TE library were combined using RepeatMasker for DNA-level repeat detection.

### Genome feature analysis and visualization

The genome features of the *P*. *stenoptera* assembly included gene density, LTR/*Gypsy* density, LTR/*Copia* density, TE density, GC content, and syntenic relationships among 16 chromosomes. The syntenic blocks within the *P*. *stenoptera* genome assembly was identified using MCScanX [85,86]. The gene density of the genome assembly was estimated using BEDTools v2.31.0 with 1000-bp windows [87]. The variation distribution of the genome features mentioned above was visualized via a Circos plot generated by TBtools v.1.120 [41].

### Population genomic analysis

We sampled a total of 28 individuals, including 9 *P*. *stenoptera*, 9 *P*. *hupehensis*, and 10 *P*. *macroptera* for whole-genome resequencing (Table S10). High-quality genomic DNA was extracted and used to construct sequencing libraries with the NEBNext Ultra DNA Library Prep Kit for Illumina (Catalog No. E7103, New England Biolabs, Ipswich, MA), followed by sequencing on Illumina NovaSeq 6000 platform (Illumina). High-quality clean reads were mapped to the *P*. *stenoptera* reference genome using BWA-MEM v0.7.15 [88]. The BAM results were marked and sorted, and then the duplicate reads were removed using SAMtools v1.3.1 [89]. SNPs were called using SAMtools v1.3.1 [89], followed by filtering based on depth, missing rate, and quality threshold using VCFtools v0.1.13 [90]. A total of 38,120,880 high-quality SNPs from 28 individuals were obtained, and 669,805 independent SNPs were selected for downstream analyses using BCFtools v1.1.2 [91] with parameters “-w 100 -n 1”. The phylogenetic tree was constructed using SVDquartets implemented in PAUP* v4.0 [92], with *J*. *mandshurica* and *C*. *paliurus* as outgroups. Genetic structures were analyzed using ADMIXTURE v1.4.0 [93] with *K* values ranging from 1 to 6. PCA was performed using EIGENSOFT v6.1.4 [94]. The mean genetic differentiation (*F*_ST_) and nucleotide diversity (*θ*π) among three species were calculated using VCFtools v0.1.13 [90]. The demographic history and effective population size were calculated using PSMC v0.6.4 [39] and SMC++ v1.15.2 [40]. The mutation rate was 2.09E−08 per site per year and generation time was 30 years [24].

### WGD events, subgenome assignment, and synteny analyses

To investigate the potential WGD events, we first identified the syntenic blocks between Chinese wingnut (*P*. *stenoptera*) and Persian walnut (*J*. *regia*), *J*. *mandshurica*, *C*. *paliurus*, *C*. *illinoinensis*, and *V*. *vinifera* using MCScanX [85] with default parameters, and then visualized the syntenic relationships via dot plots generated by JCVI [95] and TBtools [41]. Finally, we estimated the *Ks* values of syntenic blocks using KaKs_Calculator v2.0 [96] and visualized the distribution of *Ks* values using ggplot2 v3.4.2 [97].

We used the best-hit method to identify ancestral genes between *P*. *stenoptera* and *Q*. *robur*, filtered the genes based on median *Ks* < 1, and assigned them to chromosomes [22,51]. The TE analysis was performed by EDTA v2.0.0 [98]. Chromosome length was calculated using TBtools [41]. Based on the analyses mentioned above, the *P*. *stenoptera* genome was divided into two sets of subgenomes. The protein annotation was performed using eggNOG-mapper v2.1.6 [99]. The KEGG enrichment analyses were conducted using TBtools v1.120 [41].

### Comparative genomic and phylogenetic analyses

We performed comparative genomic analysis among *P*. *stenoptera*, *J*. *nigra*, *J*. *mandshurica*, *J*. *regia*, *C*. *paliurus*, *C*. *illinoinensis*, *M*. *rubra*, *Q*. *robur*, *C*. *mollissima*, *Malus domestica*, *V*. *vinifera*, *A*. *thaliana*, and *Oryza sativa* (Table S12). We conducted all-versus-all protein sequence comparisons using BLASTP [86], and then clustered gene families using OrthoMCL v2.0.9 [100]. Based on 78 single-copy orthologous genes, we constructed a maximum likelihood (ML) phylogenetic tree using RAxML v8.2.12 [101]. We analyzed gene family expansion and contraction using CAFE v5.0 [102]. We estimated the divergence time using PAML v4.5 and MCMCtree v4.5 [103].

To deeply analyze the phylogenetic relationships of *P*. *stenoptera* with other species, we also constructed a phylogenetic tree using the CDSs of chloroplast genome. The CDS sequences of chloroplast genome were extracted using PhyloSuite v1.2.2 [104], and aligned using MAFFT v.7.526 [105] with default parameters. Then, the chloroplast ML phylogenetic tree was constructed using IQ-TREE v.1.6.6 with 50,000 ultrafast bootstraps [106], and K81u was selected as the best model based on ModelFinder [107]. The Venn diagram showing the intersection of protein-coding genes among six plant species (*P*. *stenoptera*, *J*. *regia*, *C*. *paliurus*, *Q*. *robur*, *C*. *mollissima*, and *M*. *rubra*) was generated using InteractiVenn [108]. To further investigate the inconsistency between the phylogenetic trees constructed based on chloroplast and nuclear genes, we assessed the degree of hybridization or introgression using the R package “MSCquartets” [44]. Single-copy nuclear genes were obtained using OrthoFinder v2.5.4 [109], followed by the retrieval of corresponding protein sequences using HybPiper v2.1.6 [110]. Sequence alignment was performed using MAFFT v.7.5.2 [105] with the “auto” command. The resulting alignments were trimmed using trimAl v1.4.rev15 [111] with a gap threshold of “-gt 0.2”. The gene tree for each single-copy nuclear gene was constructed using IQ-TREE v.1.6.6 [106]. The quartetTable function in the R package was used to calculate the quartet count concordance factors (qcCFs) of all four-taxon partitions in the gene tree. The qcCFs were used to generate simplex diagrams through the quartetTreeTest function under Model 1 (specified species tree) and Model 3 (unspecified number of species).

### Genome-wide gene family identification

To further understand the characteristics and expression profiles of the candidate gene families related to fruit development, a genome-wide identification analysis was performed as described previously [112,113]. In brief, the protein sequences of *Arabidopsis* gene family members were used as queries to perform BLASTP (E-value < 1E−05) against the whole-genome protein sequences of six species, including *P*. *stenoptera*, *J*. *regia*, *C*. *paliurus*, *C*. *mollissima*, *Q*. *robur*, and *M*. *rubra*. Subsequently, potential candidate members were manually screened by conserved domain analysis using the Conserved Domain Database (CDD) (https://www.ncbi.nlm.nih.gov/Structure/cdd/cdd.shtml) and Pfam database (http://pfam-legacy.xfam.org/) to obtain the final gene family members.

### Scanning electron microscopy and histological analyses

For scanning electron microscopy (SEM) analysis, the floral buds of *P*. *stenoptera* were collected from four developmental stages (21 DBF, 14 DBF, 7 DBF, and 1 DBF). The samples were dissected and subjected to dehydration in a water–ethanol and ethanol–isoamyl acetate series, followed by critical-point drying in liquid CO_2_ [114]. Dried floral structures were mounted on aluminum stubs, sputter-coated with gold, and examined using a Hitachi S-3400N scanning electron microscope (Hitachi, Tokyo, Japan).

For histological analysis, winged fruits collected at 1 DAF, 15 DAF, 30 DAF, 45 DAF, and 75 DAF were fixed in formaldehyde–acetic acid–ethanol (FAA) solution [115]. The fixed materials were embedded using an embedding machine, and serial sections of 5–7 μm thickness were prepared using a Leica paraffin sectioning machine. The sections were stained with saffron and fast green, mounted with neutral gum, and observed with a Leica DMLB microscope (DMLB-B, Leica, Wetzlar, Germany).

### Transcriptomic analysis

Fresh winged fruits were collected at 1 DAF, 15 DAF, 30 DAF, 45 DAF, 75 DAF with three biological replicates and rapidly frozen in liquid nitrogen in Taiping National Forest Park, Huyi, China. All materials were subjected to total RNA extraction using TRIzol reagent (Catalog No. 15596018CN, Invitrogen, Carlsbad, MA), and the extracted RNA was tested for quality. Sequencing libraries were constructed using NEBNext Ultra II RNA Library Prep Kit for Illumina (Catalog No. E7770, New England Biolabs), and paired-end sequencing was performed on the Illumina NovaSeq 6000 platform (Illumina). The clean reads were aligned to the reference genome using Hisat v2.0.4 [75]. The obtained file was converted to BAM format by SAMtools v1.3.1 [89]. featureCounts [116] was used to calculate the gene count. The R package sva [117] was utilized to weaken the batch effect, and no significant batch effect was detected in the transcriptomic data of the five stages (Figure S16). The differential expression analysis was performed by the R package DESeq2 [118] and the threshold is |log_2_ fold change| ≥ 1 and adjusted *P* ≤ 0.05. The *K*-means analysis was performed using Metware Cloud (https://cloud.metware.cn). The WGCNA package [119] was used to analyze the weighted correlation networks between phenotypes and genes. DEGs with fragments per kilobase of exon model per million mapped fragments (FPKM) > 1 were selected as input. The soft-thresholding power was set to 17 and the genes were divided into 9 modules (Figure S17). The networks were visualized by Cytoscape v3.7.2 [120]. The phenotypic traits of fruit wings were measured using Digimizer v4.6.0 (https://www.digimizer.com/).

### qRT-PCR analysis

Total RNA was extracted from *J*. *mandshurica* and *P*. *stenoptera* fruits at five developmental stages using Plant RNA Kit (Catalog No. R6827, Omega, Guangzhou, China). Quality of total RNA was assessed based on A260/A280 ratio using NanoDrop spectrometer (KAIAO, Beijing, China). RNA was then reversely transcribed to complementary DNA (cDNA) using 5× PrimeScript RT Master Mix reverse transcriptase (Catalog No. RR036A, Takara, Kyoto, Japan). Subsequently, qRT-PCR was performed using 2× Plus SYBR real-time PCR mixture (Catalog No. PR7701, Bioteke, Wuxi, China) on Bio-Rad CFX96 real-time PCR detection system (Catalog No. CFX96, Bio-Rad, Hercules, CA). *ACTB* was used as an internal reference gene [112], and its primers were designed on Primer3Plus website (https://www.primer3plus.com). The relative expression of all genes was normalized by the 2^−ΔΔCT^ method [121]. All the primer sequences are shown in Table S20.

## Supplementary Material

qzae087_Supplementary_Data

## Data Availability

The whole-genome sequencing raw data (including Illumina short reads, PacBio long reads, and Hi-C interaction reads), the transcriptomic raw data, and the whole-genome resequencing raw data generated in this study have been deposited in the Genome Sequence Archive [122] at the National Genomics Data Center (NGDC), Beijing Institute of Genomics (BIG), Chinese Academy of Sciences (CAS) / China National Center for Bioinformation (CNCB) (GSA: CRA019683 for *J*. *mandshurica* transcriptomic raw data; CRA019682 for *P*. *stenoptera* whole-genome sequencing, transcriptomic, and whole-genome resequencing raw data), and are publicly accessible at https://ngdc.cncb.ac.cn/gsa. The genome assemblies and annotations have been deposited in the Genome Warehouse [123] at the NGDC, BIG, CAS / CNCB (GWH: GWHFGFO00000000.1), and are publicly accessible at https://ngdc.cncb.ac.cn/gwh.

## CRediT author statement

**Fangdong Geng:** Investigation, Resources, Methodology, Formal analysis, Visualization, Writing – original draft, Writing – review & editing. **Xuedong Zhang:** Investigation, Methodology, Formal analysis, Visualization, Writing – review & editing. **Jiayu Ma:** Methodology, Formal analysis, Visualization, Writing – review & editing. **Hengzhao Liu:** Methodology, Visualization. **Hang Ye:** Methodology, Visualization. **Fan Hao:** Formal analysis, Visualization. **Miaoqing Liu:** Methodology, Formal analysis. **Meng Dang:** Formal analysis. **Huijuan Zhou:** Conceptualization, Methodology, Writing – original draft, Writing – review & editing. **Mengdi Li:** Conceptualization, Methodology, Writing – original draft, Writing – review & editing. **Peng Zhao:** Conceptualization, Methodology, Resources, Writing – original draft, Writing – review & editing. All authors have read and approved the final manuscript.
