# Supplementary material for: Genome Assembly and Winged Fruit Gene Regulation of Chinese Wingnut: Insights from Genomic and Transcriptomic Analyses
Source: Genomics Proteomics Bioinformatics. 2024 Dec 12;22(6):qzae087. doi: 10.1093/gpbjnl/qzae087 (PMC12043009; doi:10.1093/gpbjnl/qzae087)
Supplement: qzae087_Supplementary_Data [file qzae087_supplementary_data.zip › Figure S5.pdf]

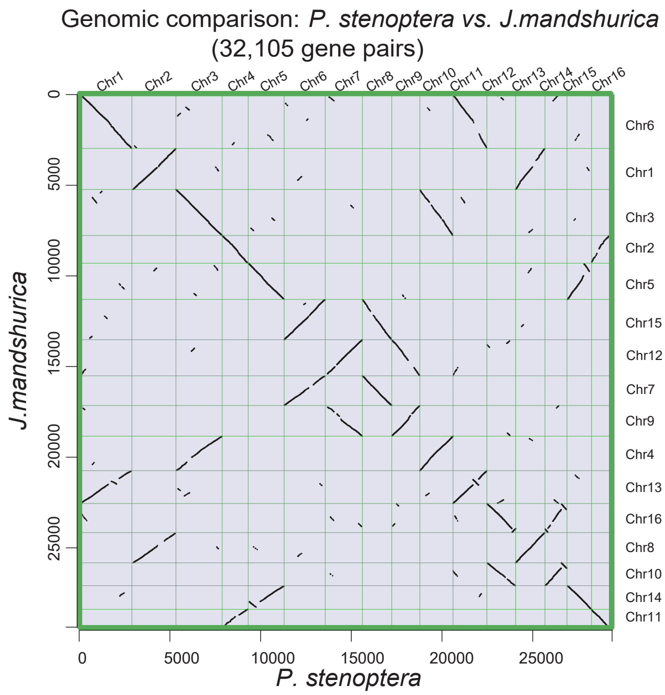
Genomic comparison: P. stenoptera vs. J.mandshurica
(32,105 gene pairs)
Chr1 Chr2 Chr3 Chr4 Chr5 Chr6 Chr7 Chr8 Chr9 Chr10 Chr11 Chr12 Chr13 Chr14 Chr15 Chr16
Chr6 Chr1 Chr3 Chr2 Chr5 Chr15 Chr12 Chr7 Chr9 Chr4 Chr13 Chr16 Chr8 Chr10 Chr14 Chr11
0 5000 10000 15000 20000 25000
J.mandshurica
P. stenoptera

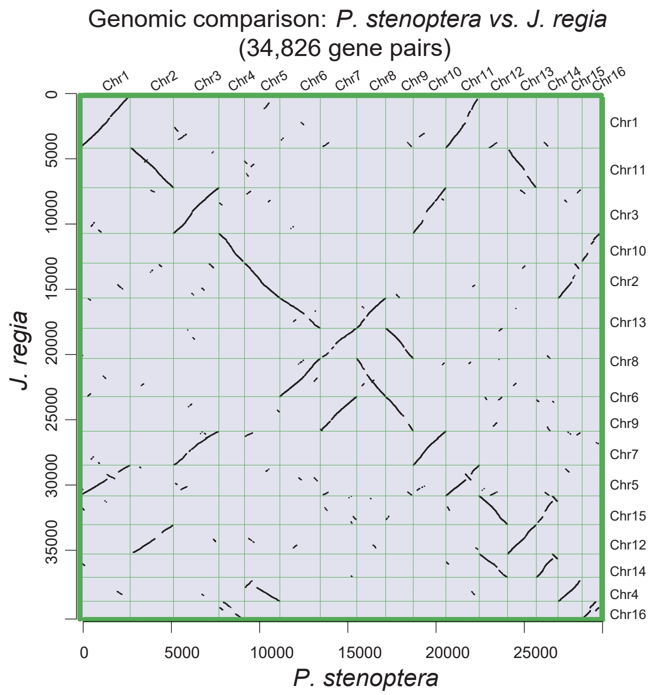
Genomic comparison: P. stenoptera vs. J. regia
(34,826 gene pairs)
Chr1 Chr2 Chr3 Chr4 Chr5 Chr6 Chr7 Chr8 Chr9 Chr10 Chr11 Chr12 Chr13 Chr14 Chr15 Chr16
Chr1 Chr11 Chr3 Chr10 Chr2 Chr13 Chr8 Chr6 Chr9 Chr7 Chr5 Chr15 Chr12 Chr14 Chr4 Chr16
0 5000 10000 15000 20000 25000 30000 35000
J. regia
P. stenoptera

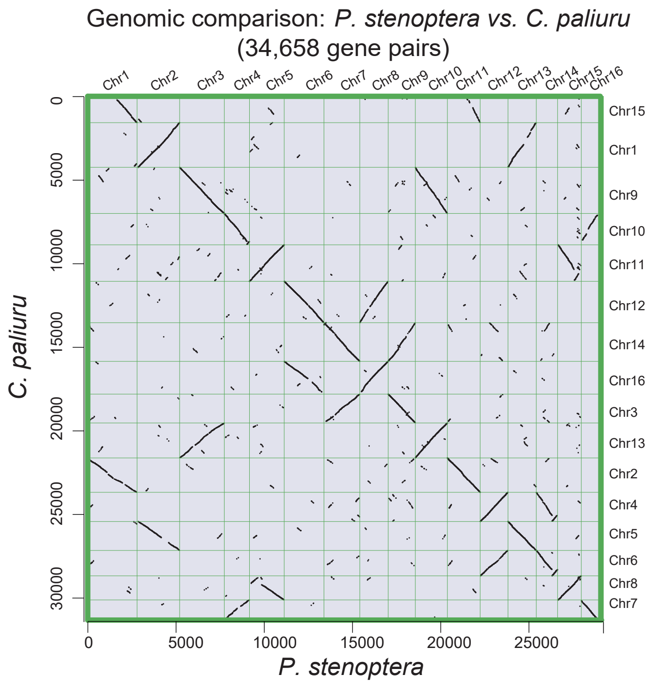
Genomic comparison: P. stenoptera vs. C. paliuru
(34,658 gene pairs)
Chr1 Chr2 Chr3 Chr4 Chr5 Chr6 Chr7 Chr8 Chr9 Chr10 Chr11 Chr12 Chr13 Chr14 Chr15 Chr16
Chr15 Chr1 Chr9 Chr10 Chr11 Chr12 Chr14 Chr16 Chr3 Chr13 Chr2 Chr4 Chr5 Chr6 Chr8 Chr7
0 5000 10000 15000 20000 25000 30000
C. paliuru
P. stenoptera

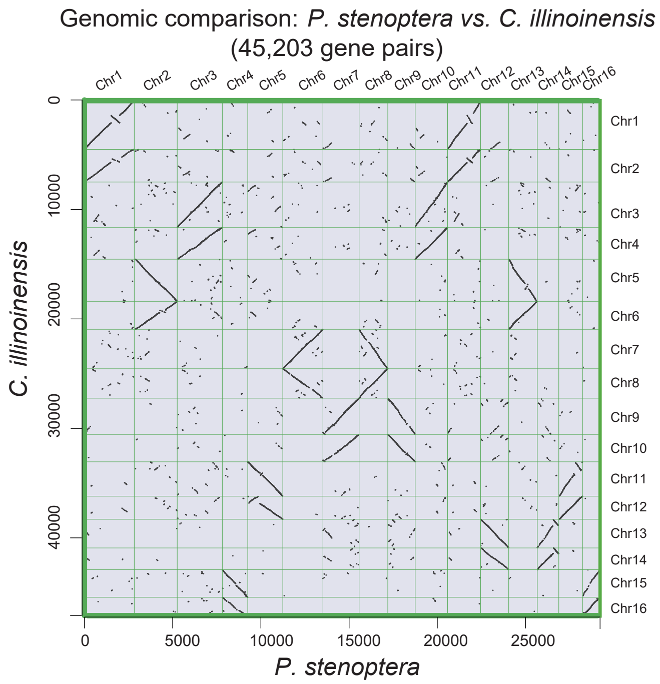
Genomic comparison: P. stenoptera vs. C. illinoinensis
(45,203 gene pairs)
Chr1 Chr2 Chr3 Chr4 Chr5 Chr6 Chr7 Chr8 Chr9 Chr10 Chr11 Chr12 Chr13 Chr14 Chr15 Chr16
Chr1 Chr2 Chr3 Chr4 Chr5 Chr6 Chr7 Chr8 Chr9 Chr10 Chr11 Chr12 Chr13 Chr14 Chr15 Chr16
0 5000 10000 15000 20000 25000 30000 40000
C. illinoinensis
P. stenoptera

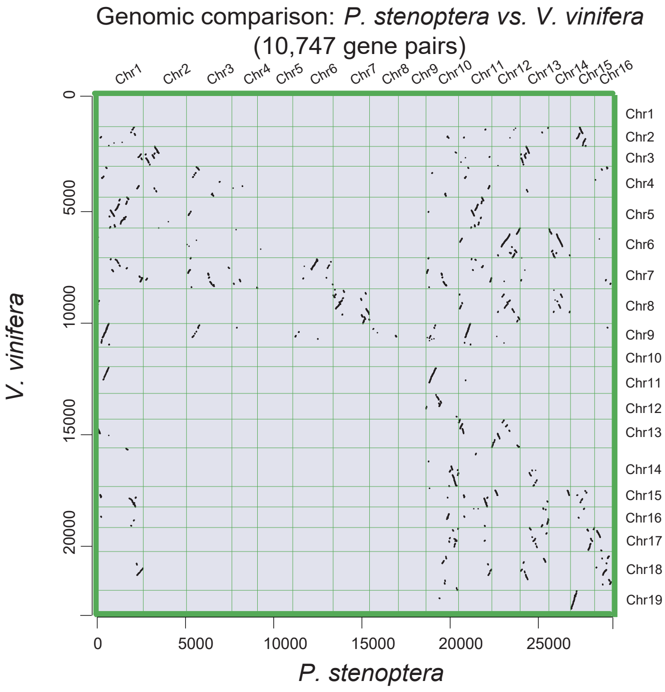
Genomic comparison: P. stenoptera vs. V. vinifera
(10,747 gene pairs)
Chr1 Chr2 Chr3 Chr4 Chr5 Chr6 Chr7 Chr8 Chr9 Chr10 Chr11 Chr12 Chr13 Chr14 Chr15 Chr16
Chr1 Chr2 Chr3 Chr4 Chr5 Chr6 Chr7 Chr8 Chr9 Chr10 Chr11 Chr12 Chr13 Chr14 Chr15 Chr16 Chr17 Chr18 Chr19
0 5000 10000 15000 20000 25000
V. vinifera
P. stenoptera
