# Supplementary material for: Genome Assembly and Winged Fruit Gene Regulation of Chinese Wingnut: Insights from Genomic and Transcriptomic Analyses
Source: Genomics Proteomics Bioinformatics. 2024 Dec 12;22(6):qzae087. doi: 10.1093/gpbjnl/qzae087 (PMC12043009; doi:10.1093/gpbjnl/qzae087)
Supplement: qzae087_Supplementary_Data [file qzae087_supplementary_data.zip › Supplementary material captions.docx]

**Supplementary material**

**Figure S1 Genome size estimation by 17-*k*-mer analysis of *P*. *stenoptera***

**Figure S2 Hi-C interaction heatmap between 16 chromosomes of the *P*. *stenoptera* genome**

**Figure S3 Genome collinearity between three *P*. *stenoptera* assemblies**

Reference genome was our *P*. *stenoptera* assembly in this study.

**Figure S4 The CV errors from 28 populations of three *Pterocarya* species**

Values at *K* = 3 indicate three clusters as the most appropriate option. CV, cross-validation.

**Figure S5 Assembled chromosome dot-maps between *P*. *stenoptera* and different Juglandaceae species and *Vitis vinifera***

The black dots indicate homoeologous chromosomes within different genome.

**Figure S6 Dot-maps between different chromosomes of *P*. *stenoptera***

The red dots indicate homoeologous chromosomes within different genome.

**Figure S7 The paralogous syntenic relationships among sixteen chromosomes of *P*. *stenoptera***

There are eight main duplication sub genomes, and the chromosome pairs are as follows: Chr1 and Chr11, Chr2 and Chr13, Chr3 and Chr10, Chr4 and Chr16, Chr5 and Chr15, Chr6 and Chr8, Chr7 and Chr9, and Chr12 and Chr14.

**Figure S8 Dot-maps between different paralogous chromosomes of *P*. *stenoptera***

The black dots indicate homoeologous chromosomes within different chromosomes.

**Figure S9 Basic numerical statistics between two sets of subgenomes**

**A.** The number of TEs between different chromosomes. **B.** The number of genes between different chromosomes. **C.** The length of chromosomes between different subgenomes. Blue represents the DS and red represents the SS.

**Figure S10**  **The KEGG enrichment analysis in the SS of *P*. *stenoptera* assembly**

**Figure S11 Simplex plots of qcCFs under the MSC model and the T1 (specific species tree topology hypothesized) and T3 model (no specific species tree topology hypothesized) using the R package MSCquartets**

Red triangles in the plot represent rejection of the MSC model and indicate gene tree discord perhaps caused by introgression; blue circles represent a failure to reject the null hypothesis of MSC model. **A**. Five species (*P*. *stenoptera*, *C*. *paliurus*, *J*. *mandshurica*, *C*. *mollissima*, and *M*. *rubra*) were used to detect gene tree conflicts among *P*. *stenopetera*, *C*. *paliurus*, and *J*. *mandshurica*. **B**. Five species (*J*. *regia*, *J*. *nigra*, *J*. *mandshurica*, *C*. *mollissima*, and *M*. *rubra*) were used to detect gene tree conflicts among *J*. *regia*, *J*. *nigra*, and *J*. *mandshurica*. qcCFs, the quartet count concordance factors; MSC, multispecies coalescent.

**Figure S12 *LOX* gene family analysis**

**A.** The ML phylogenetic tree of *LOX* protein of *A*. *thaliana*, *P*. *stenoptera*, *J*. *mandshurica*, and *C*. *paliurus*. **B.** Chromosomal distribution of *LOXs*. Yellow, green, and blue chromosomal indicate *P*. *stenoptera*, *J*. *mandshurica*, and *C*. *paliurus*, respectively. **C.** Gene structures of *LOX*s in *P*. *stenoptera*, *J*. *mandshurica*, and *C*. *paliurus.* Green boxes indicated exons, and gray lines indicate introns. **D.** Genome-wide synteny analysis for *LOX*s among *P*. *stenoptera*, *J*. *mandshurica*, and *C*. *paliurus.* Blue lines indicate orthologous gene pairs, and red lines indicate paralogous gene pairs.

**Figure S13 Morphology of fruit development and expression analysis of *LOX* genes in *P*. *stenoptera* and *J*. *mandshurica***

**A.** Fruit morphology at five developmental stages of the *J*. *mandshurica*. **B.** FPKM and qRT-PCR values of the *evm.model.Chr12.1795* gene at five developmental stages in *P*. *stenoptera*. **C.** FPKM and qRT-PCR values of the *Jma000017058* gene at five developmental stages in *J*. *mandshurica.* qRT-PCR, Quantitative Real-time Polymerase Chain Reaction.

**Figure S14 Repeatability verification of fifteen samples from five periods**

**A.** Correlation heat map of different stages of *P*. *stenoptera.* **B.** PCA plots of different stages of *P*. *stenoptera.*

**Figure S15 KEGG enrichment map of genes of cluster 1, 5, and 3 by K-means**

Enrich factor of abscissa indicates degree of enrichment. The color and size of the points represent the range of the *P* value and the number of differentially expressed genes enriched, respectively.

**Figure S16 PCA analysis of batch effects**

**A.** PCA plot before removal of batch effects. **B.** PCA plot after removal of batch effects.

**Figure S17 Weighted correlation network analysis of differentially expressed genes**

**A.** Scale independence and mean connectivity. The soft threshold is selected according to the R^2^. The soft threshold is 17, when R^2^ is first greater than 0.85. **B.** Gene dendrogram and module colors. The genes are divided into nine main modules.

**Table S1 Summary of sequencing data of *Pterocarya stenoptera***

**Table S2 Summary of the genome assembly of *Pterocarya stenoptera* based on Hi-C sequencing data**

**Table S3 Anchored rates of *Pterocarya stenoptera* genome**

**Table S4 Statistical summary of the sizes of the chromosomes of *Pterocarya stenoptera* genome assembly**

**Table S5 Results of the BUSCO assessment of single-copy genes and proteins of the *Pterocarya stenoptera***

**Table S6 Assembly statistics for three *Pterocarya stenoptera* genomes**

**Table S7 Repeat sequence annotation of the *Pterocarya stenoptera* genome**

**Table S8 Statistical summary of the annotation of the *Pterocarya stenoptera* genome using six databases (Swiss-Prot, NR, KEGG, InterPro, GO, and Pfam)**

**Table S9 Abundance and size of non-coding RNA in *Pterocarya stenoptera* genome**

**Table S10 Statistical summary of whole-genome resequencing from 28 individuals mapped onto the *Pterocarya stenoptera* genome assembly**

**Table S11 Distribution of ancestral genes in chromosomes**

**Table S12 Summary of genome comparisons and clusters of gene families in 13 species**

**Table S13 FPKM statistics of lipid metabolism genes in different stages of fruit wing development**

**Table S14 Estimated *K*_a_/*K*_s_ ratios of gene pairs**

**Table S15 The tissues, collection date, and RNA sequencing data of samples used for transcriptome analysis of *Pterocarya stenoptera* and *Juglans mandshurica* in this study**

**Table S16 FPKM statistics of starch and sucrose metabolism genes in different stages of fruit wing development**

**Table S17 Phenotypic data of *Pterocarya stenoptera* from WGCNA**

**Table S18 FPKM statistics of *SUC2*, *ARF7*, lignin biosynthesis-related genes, and MADS-box genes in different stages of fruit wing development**

**Table S19 The tissues, collection date, and RNA sequencing data of samples used for genome annotation of *Pterocarya stenoptera* in this study**

**Table S20 Primers for the qRT-PCR experiment**
