# Supplementary figures and images for: Genome Assembly and Winged Fruit Gene Regulation of Chinese Wingnut: Insights from Genomic and Transcriptomic Analyses
Source: Genomics Proteomics Bioinformatics. 2024 Dec 12;22(6):qzae087. doi: 10.1093/gpbjnl/qzae087 (PMC12043009; doi:10.1093/gpbjnl/qzae087)

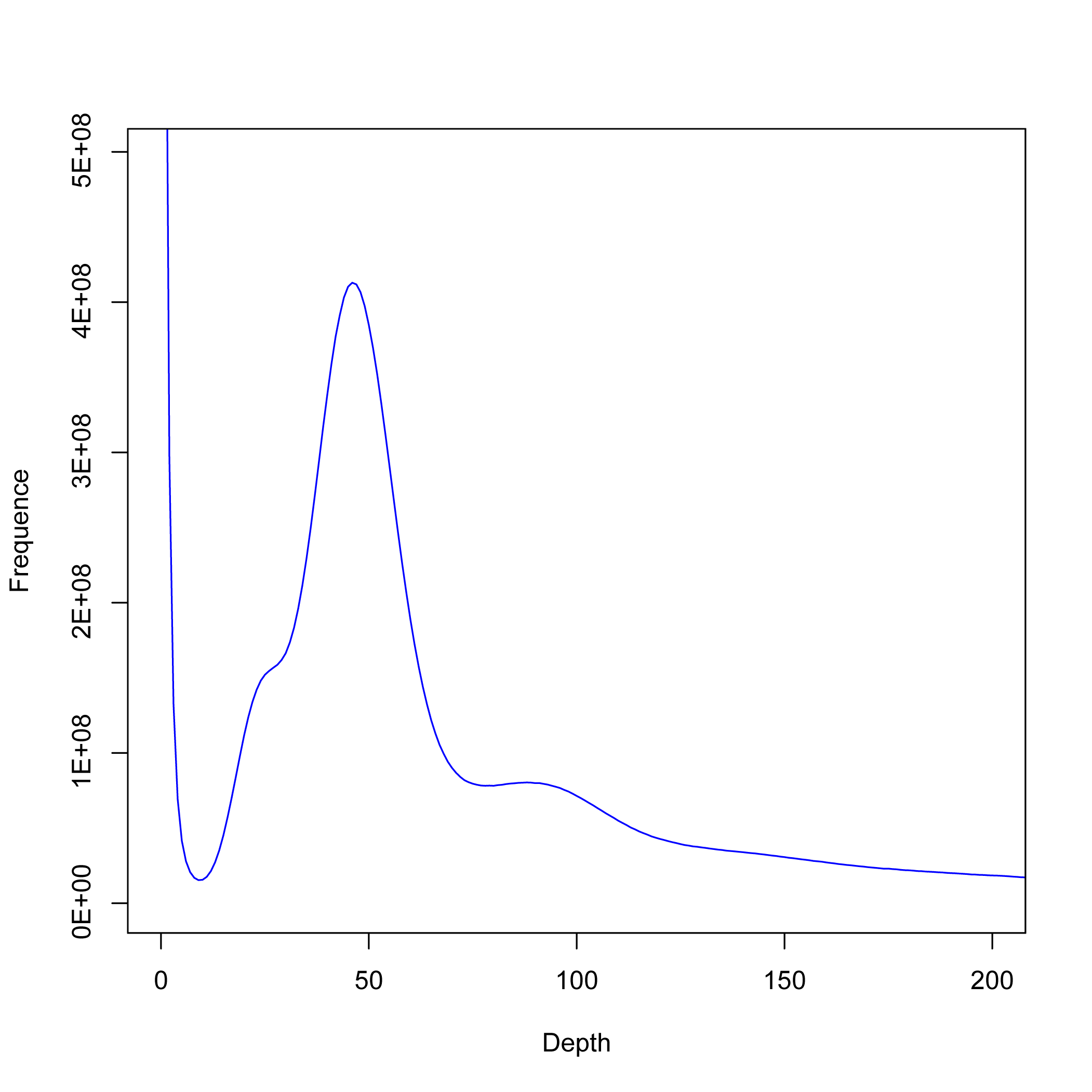

Supplement: qzae087_Supplementary_Data [file qzae087_supplementary_data.zip › Figure S1.tif]

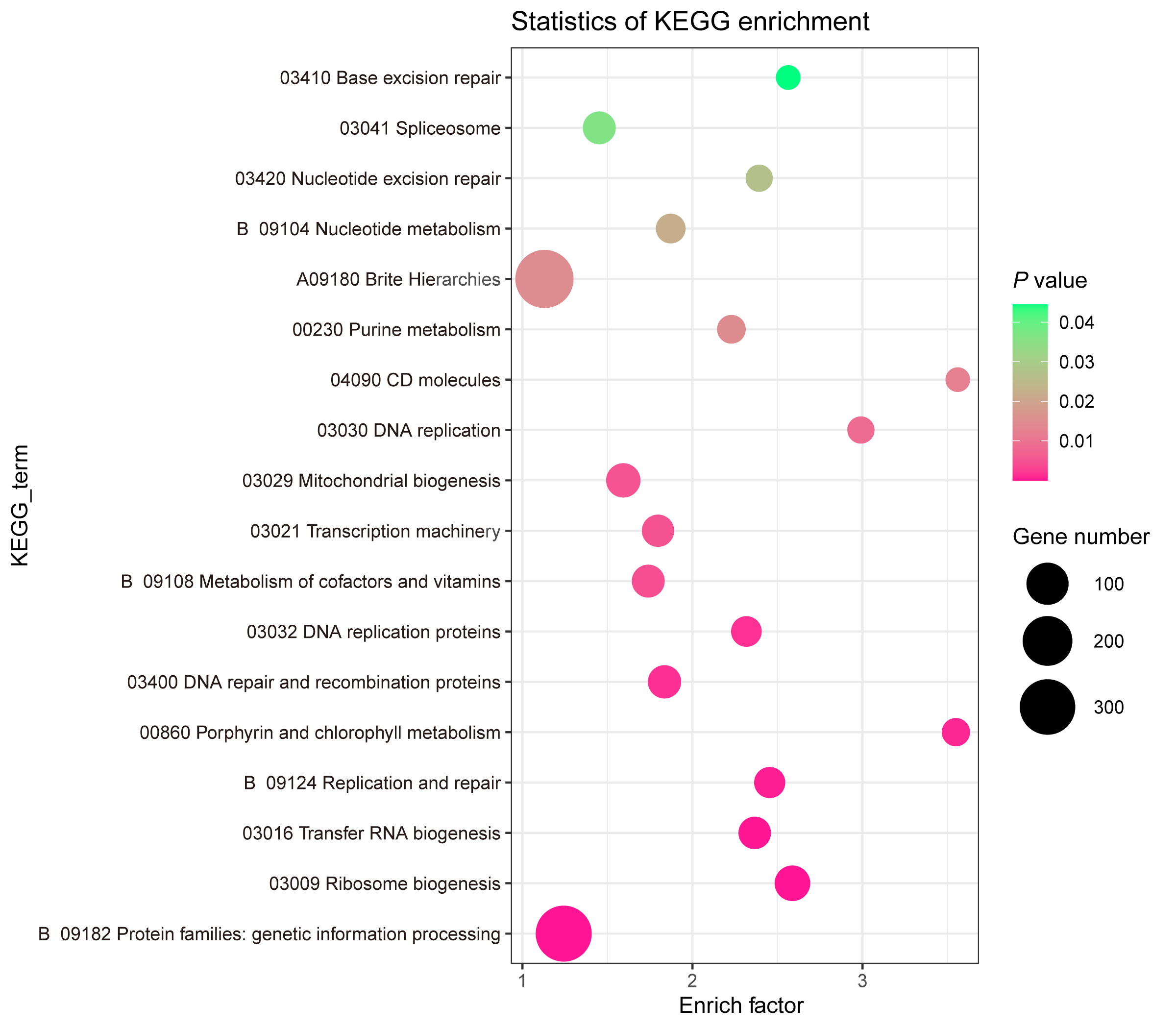

Supplement: qzae087_Supplementary_Data [file qzae087_supplementary_data.zip › Figure S10.tif]

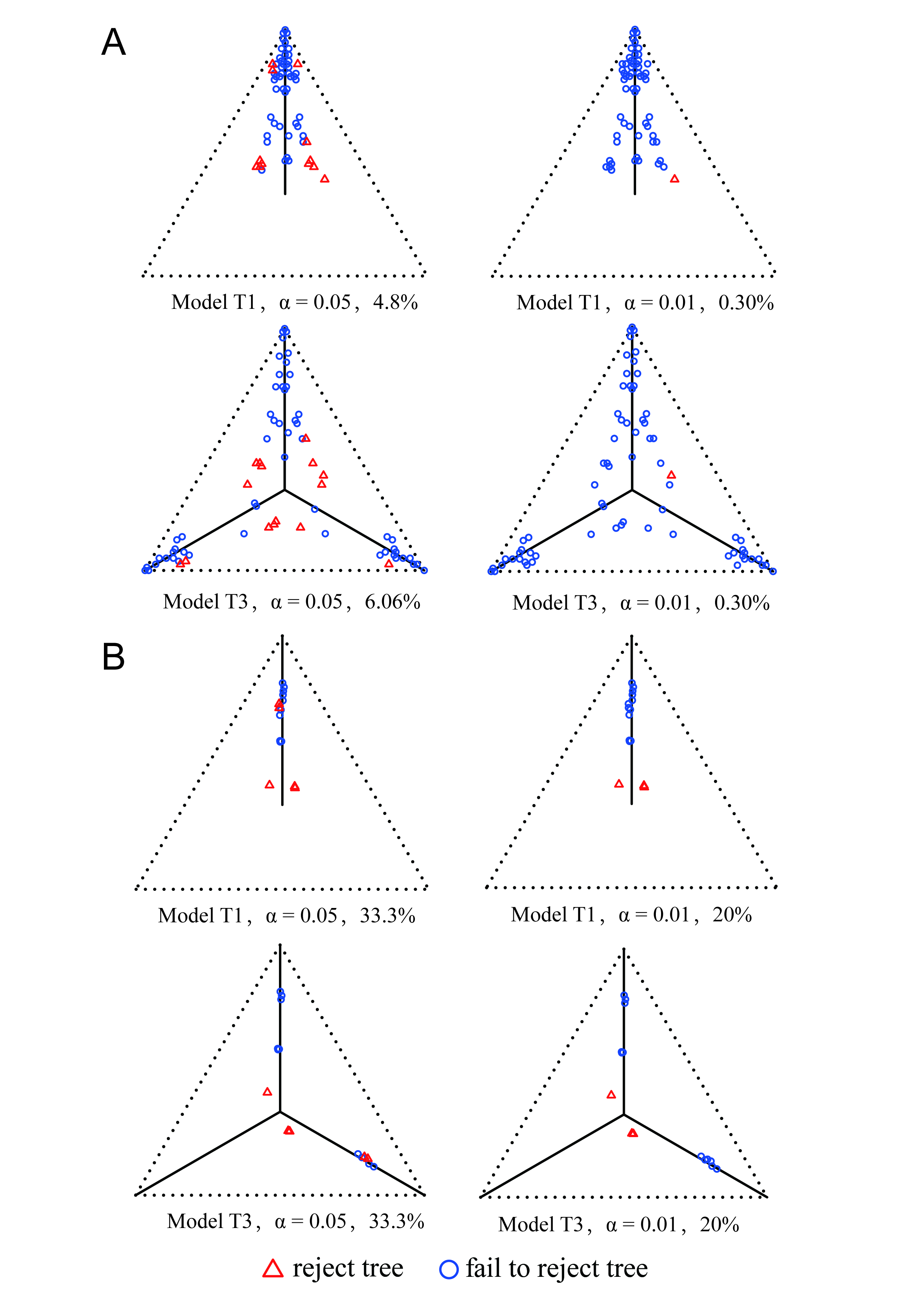

Supplement: qzae087_Supplementary_Data [file qzae087_supplementary_data.zip › Figure S11.tif]

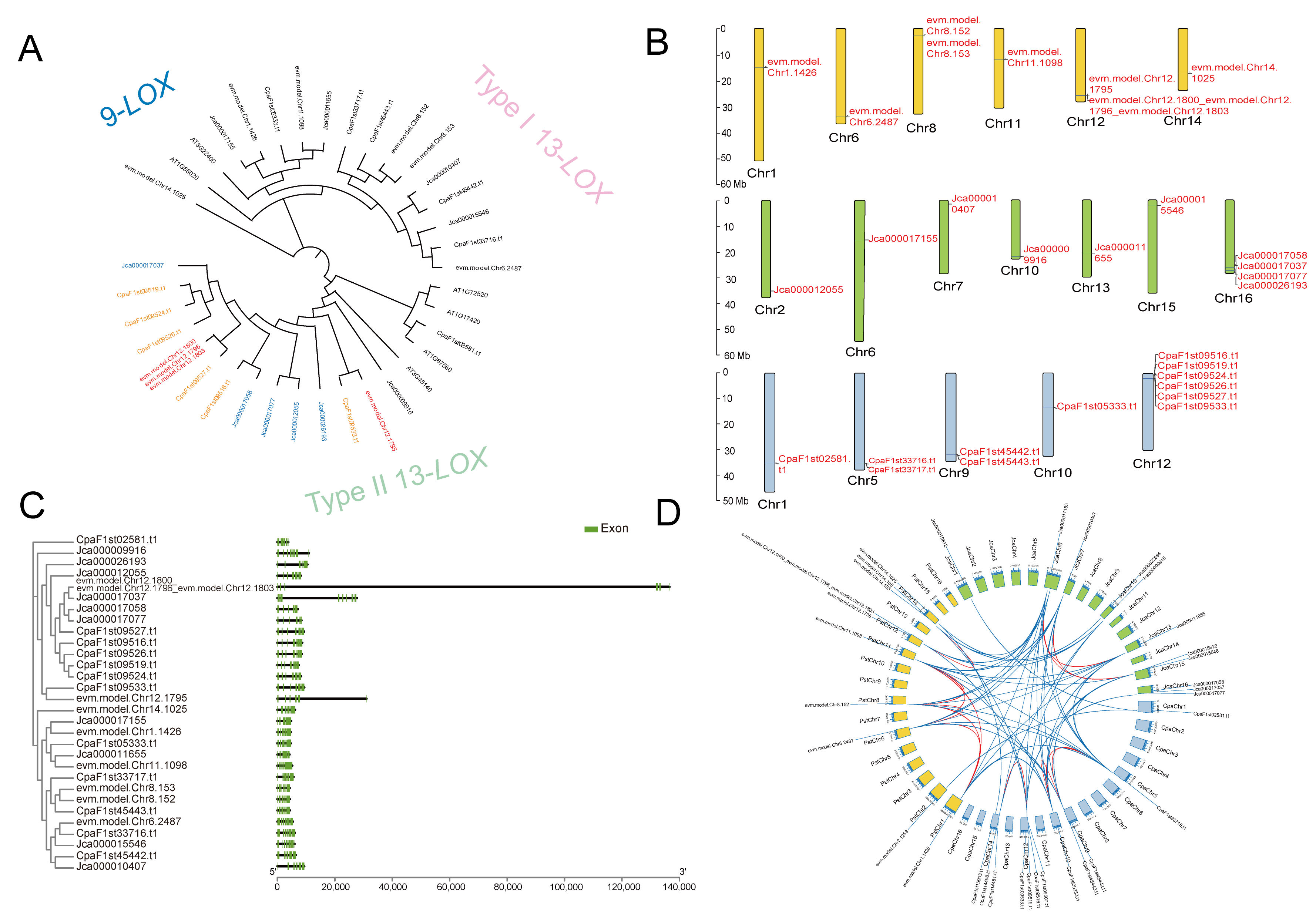

Supplement: qzae087_Supplementary_Data [file qzae087_supplementary_data.zip › Figure S12.tif]

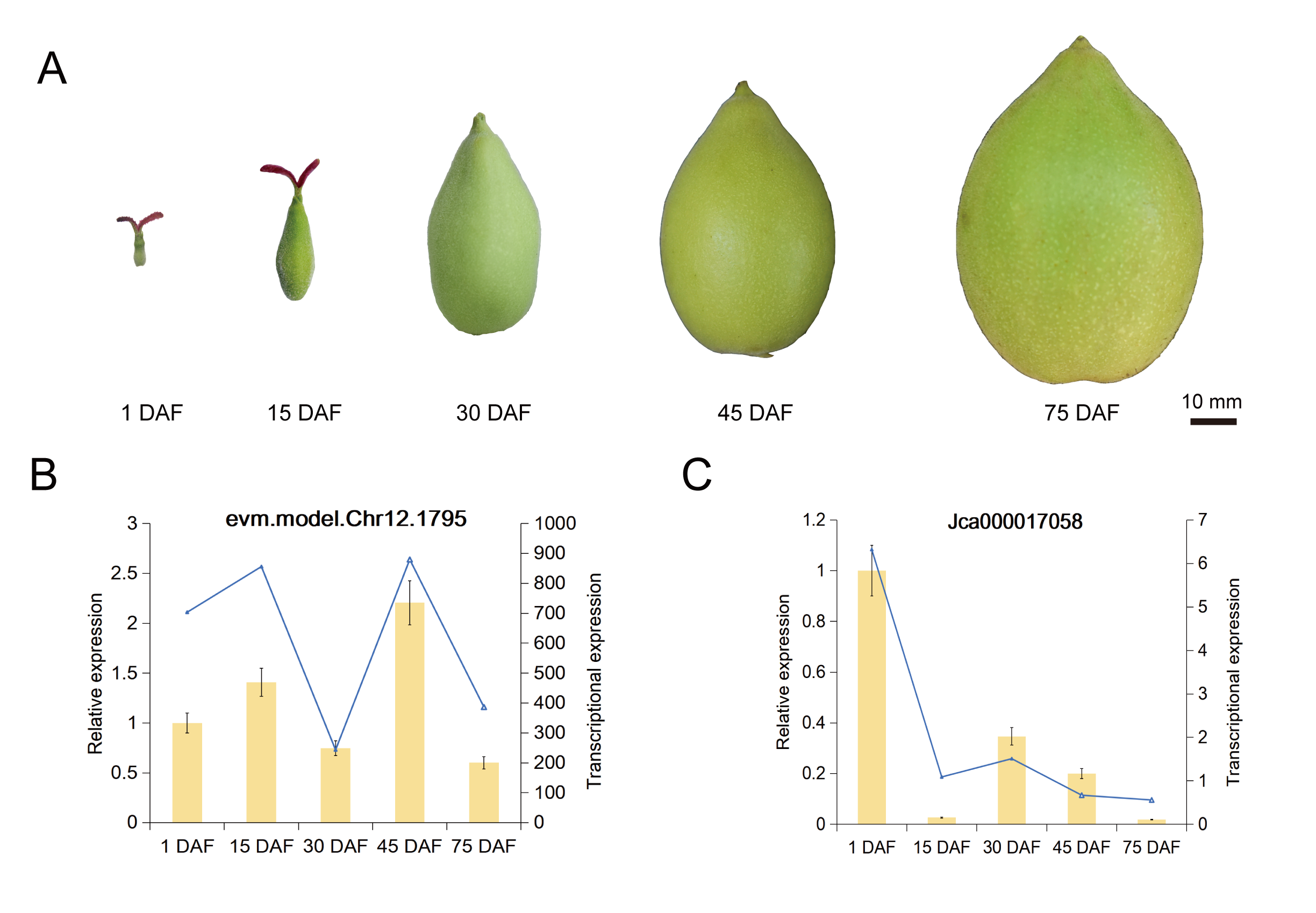

Supplement: qzae087_Supplementary_Data [file qzae087_supplementary_data.zip › Figure S13.tif]

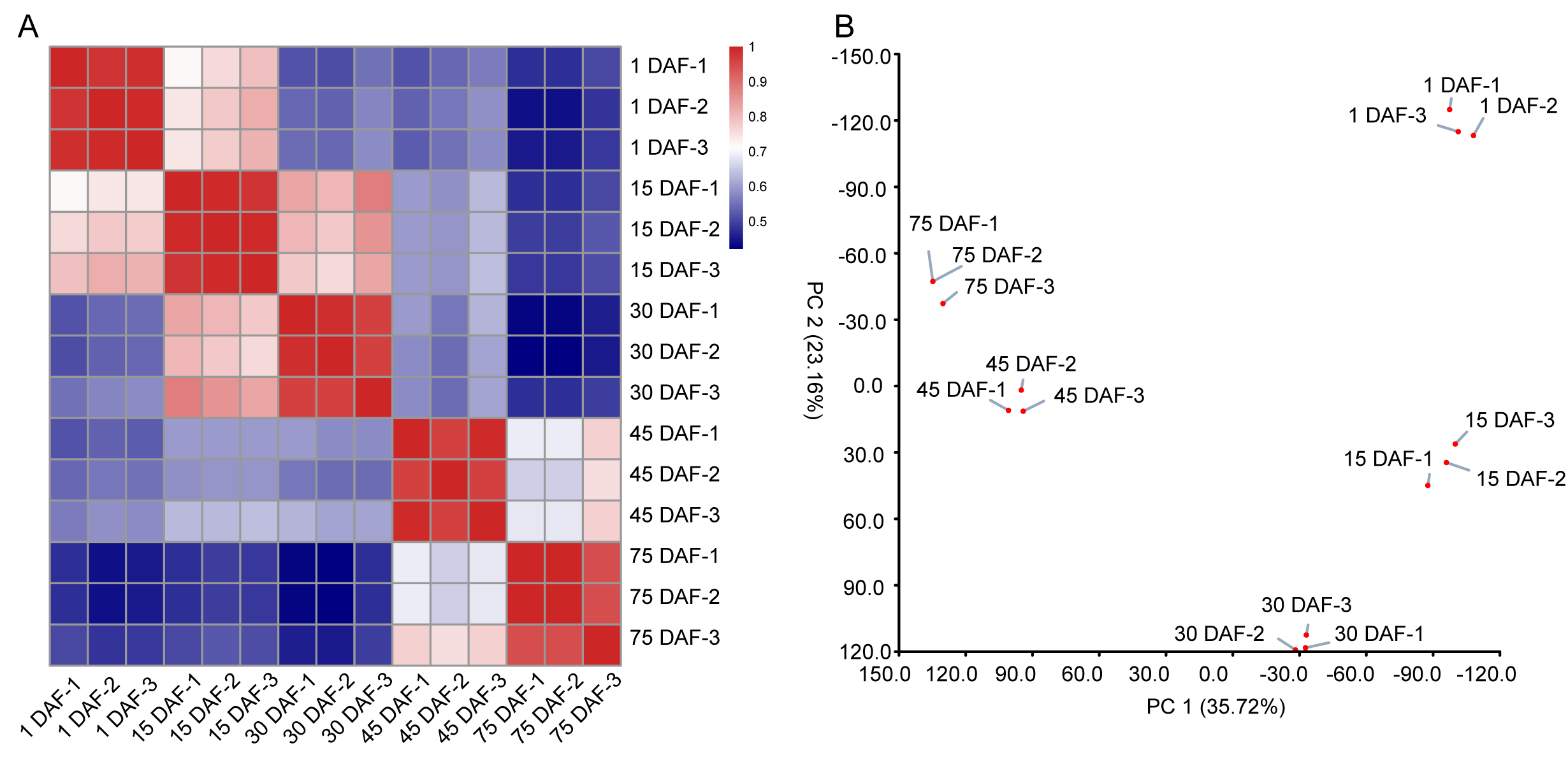

Supplement: qzae087_Supplementary_Data [file qzae087_supplementary_data.zip › Figure S14.tif]

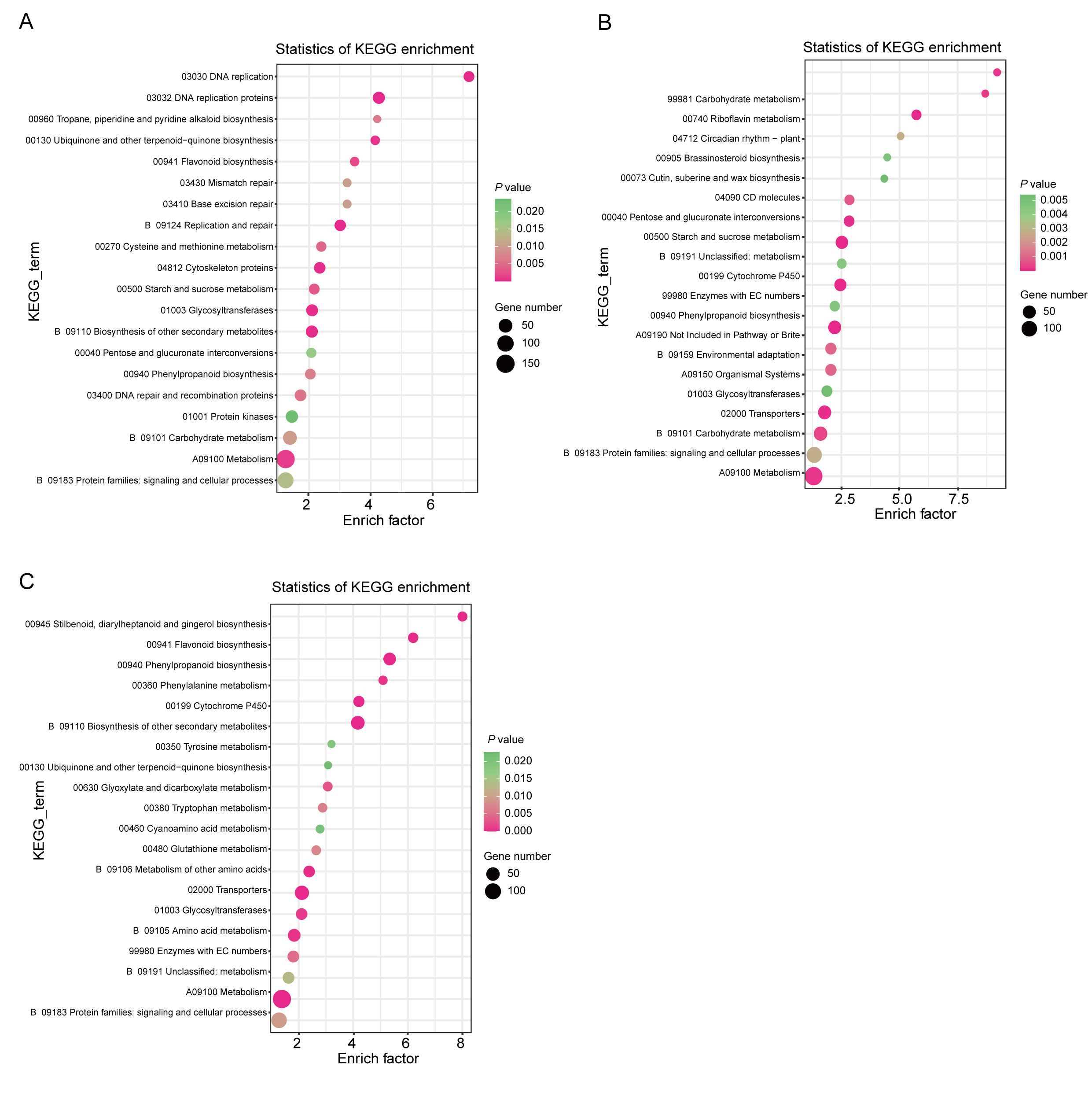

Supplement: qzae087_Supplementary_Data [file qzae087_supplementary_data.zip › Figure S15.tif]

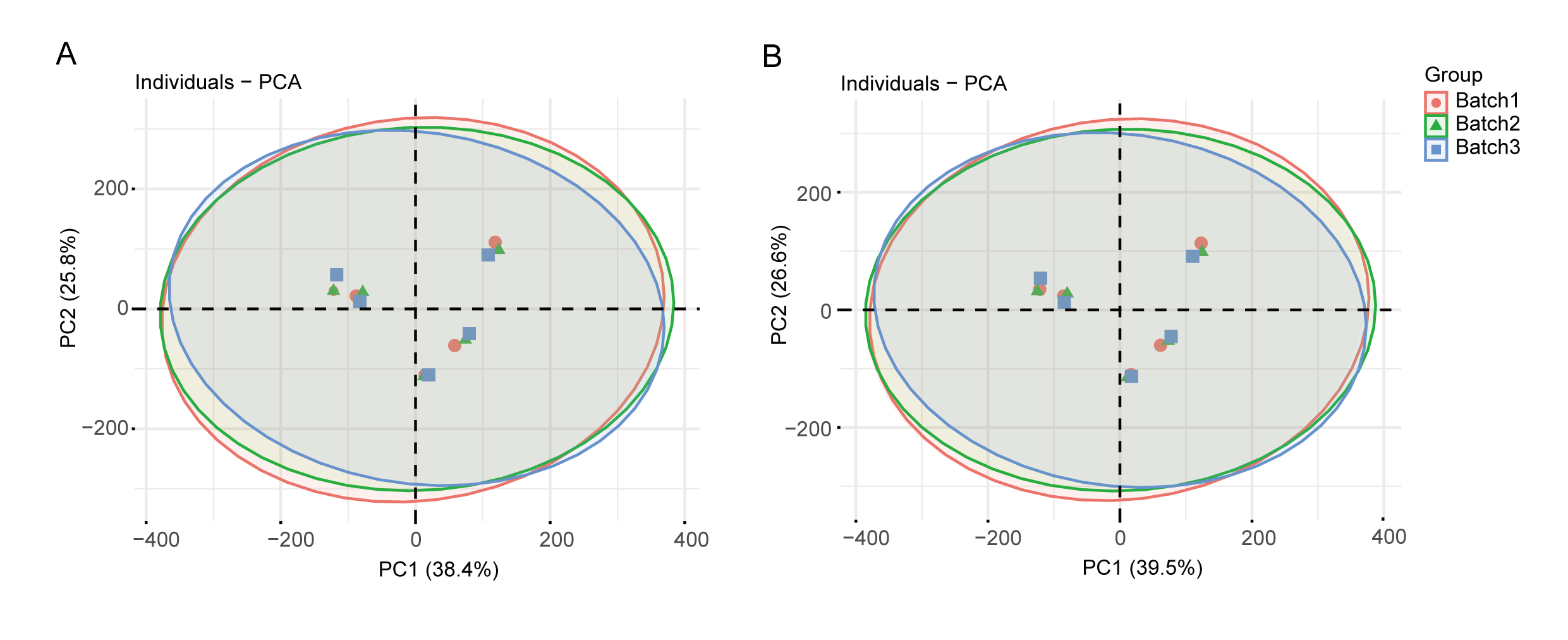

Supplement: qzae087_Supplementary_Data [file qzae087_supplementary_data.zip › Figure S16.tif]

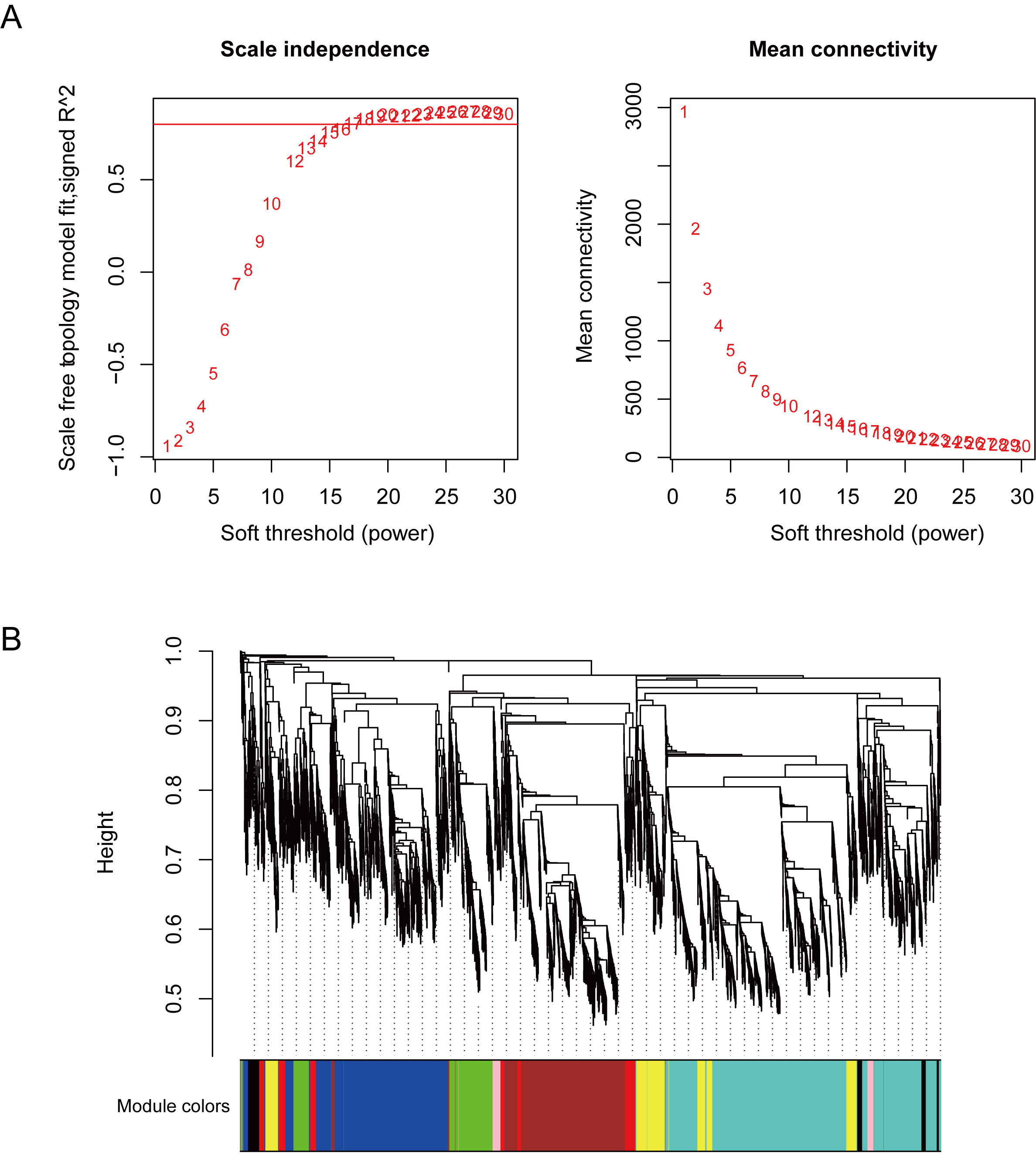

Supplement: qzae087_Supplementary_Data [file qzae087_supplementary_data.zip › Figure S17.tif]

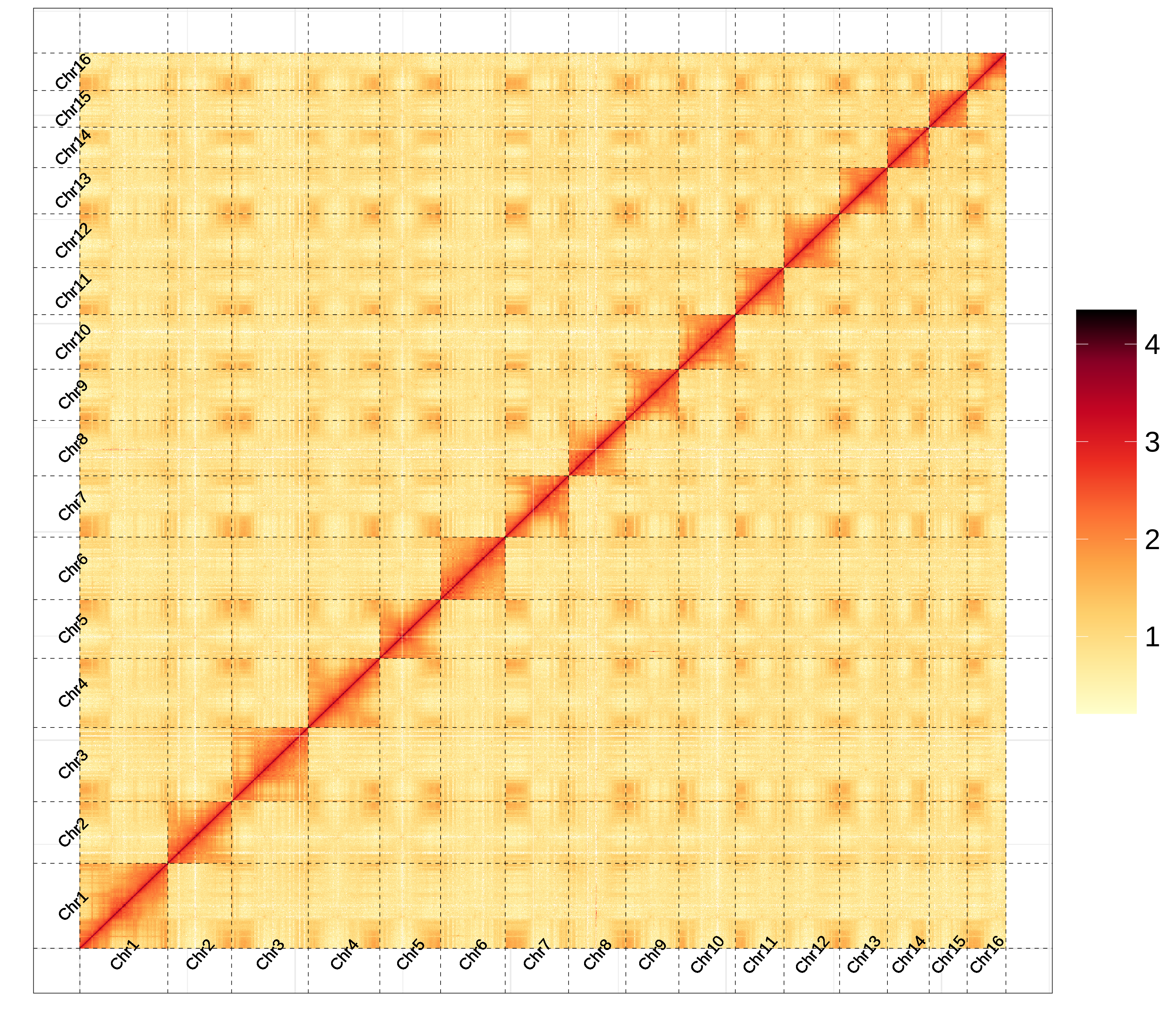

Supplement: qzae087_Supplementary_Data [file qzae087_supplementary_data.zip › Figure S2.tif]

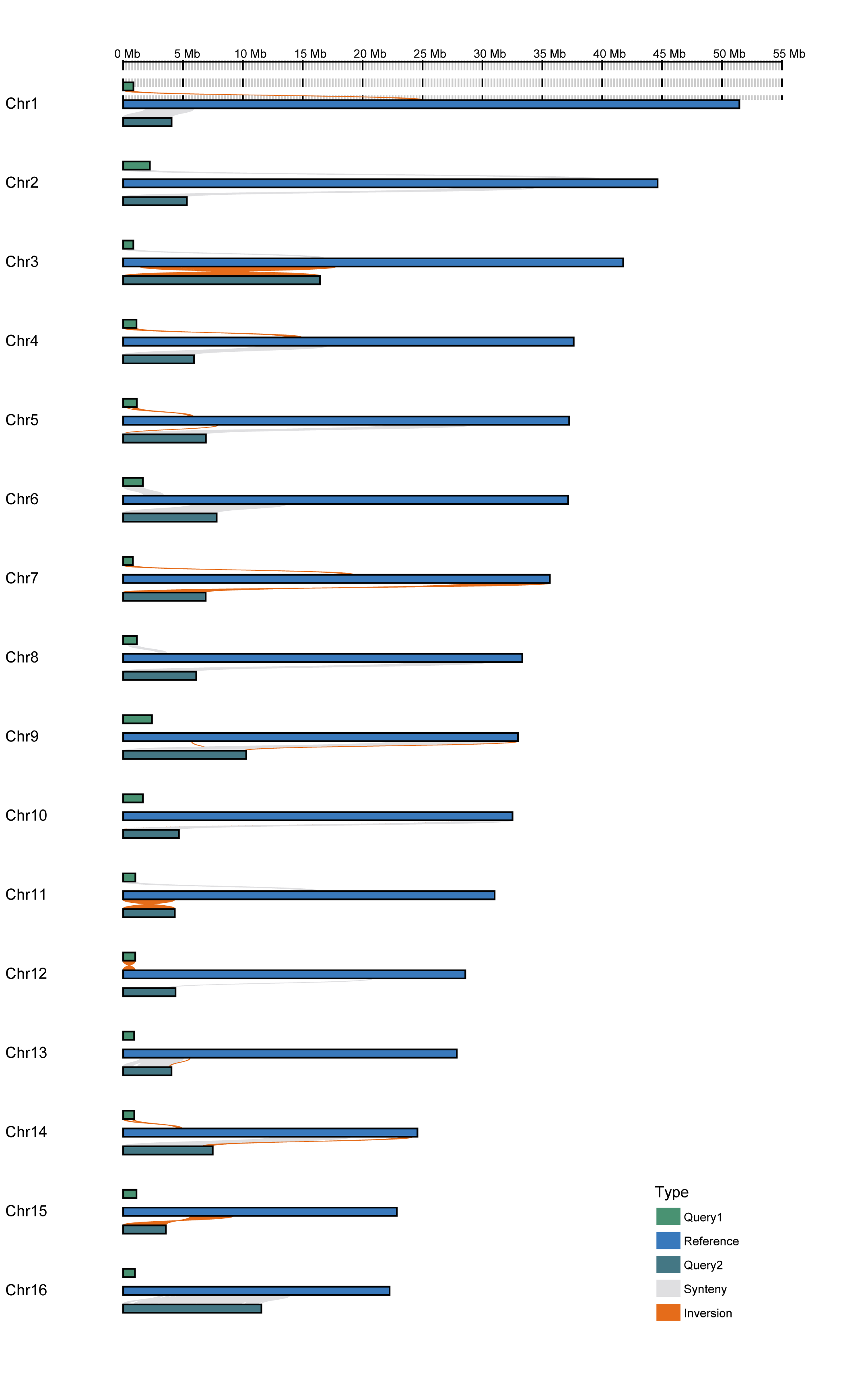

Supplement: qzae087_Supplementary_Data [file qzae087_supplementary_data.zip › Figure S3.tif]

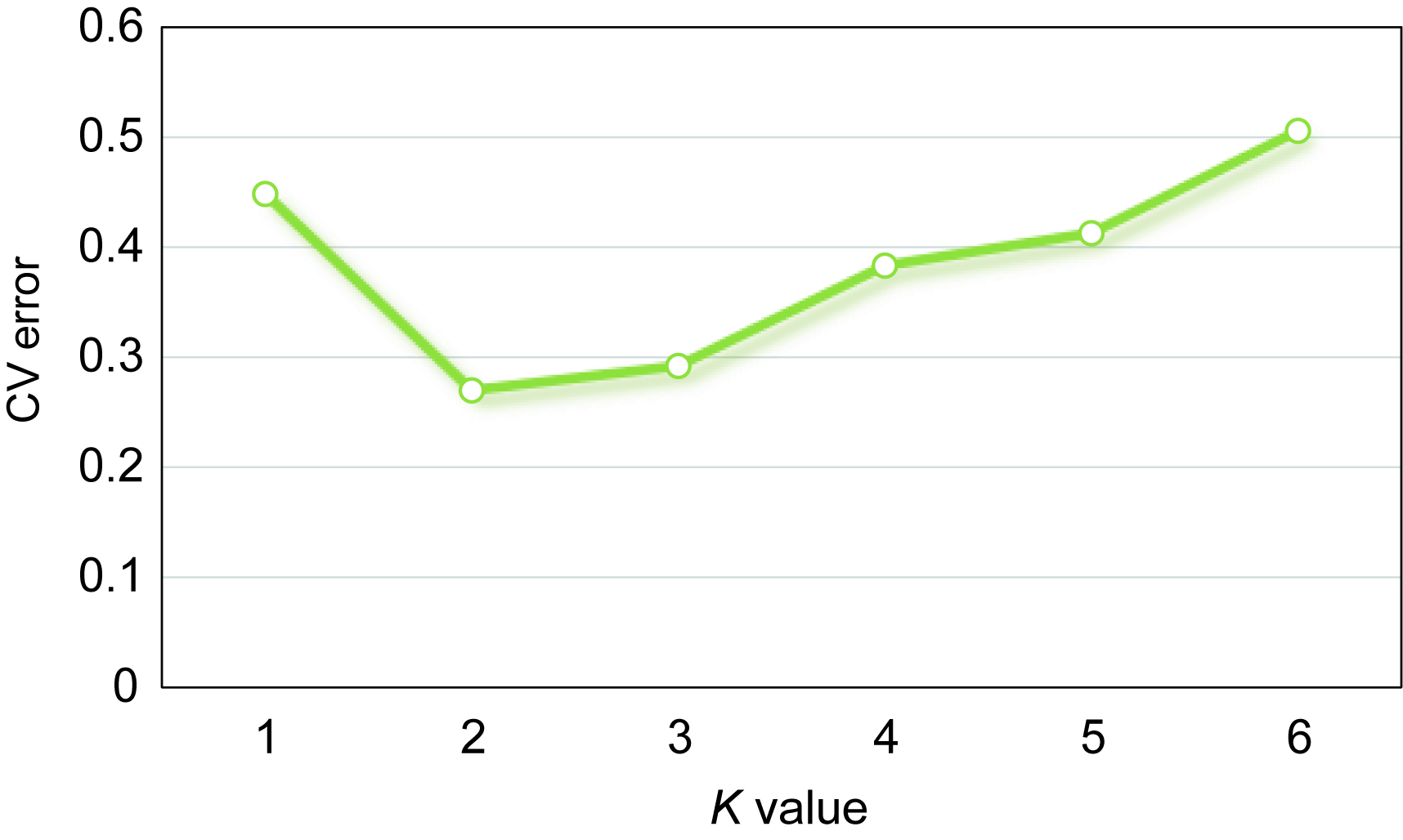

Supplement: qzae087_Supplementary_Data [file qzae087_supplementary_data.zip › Figure S4.tif]

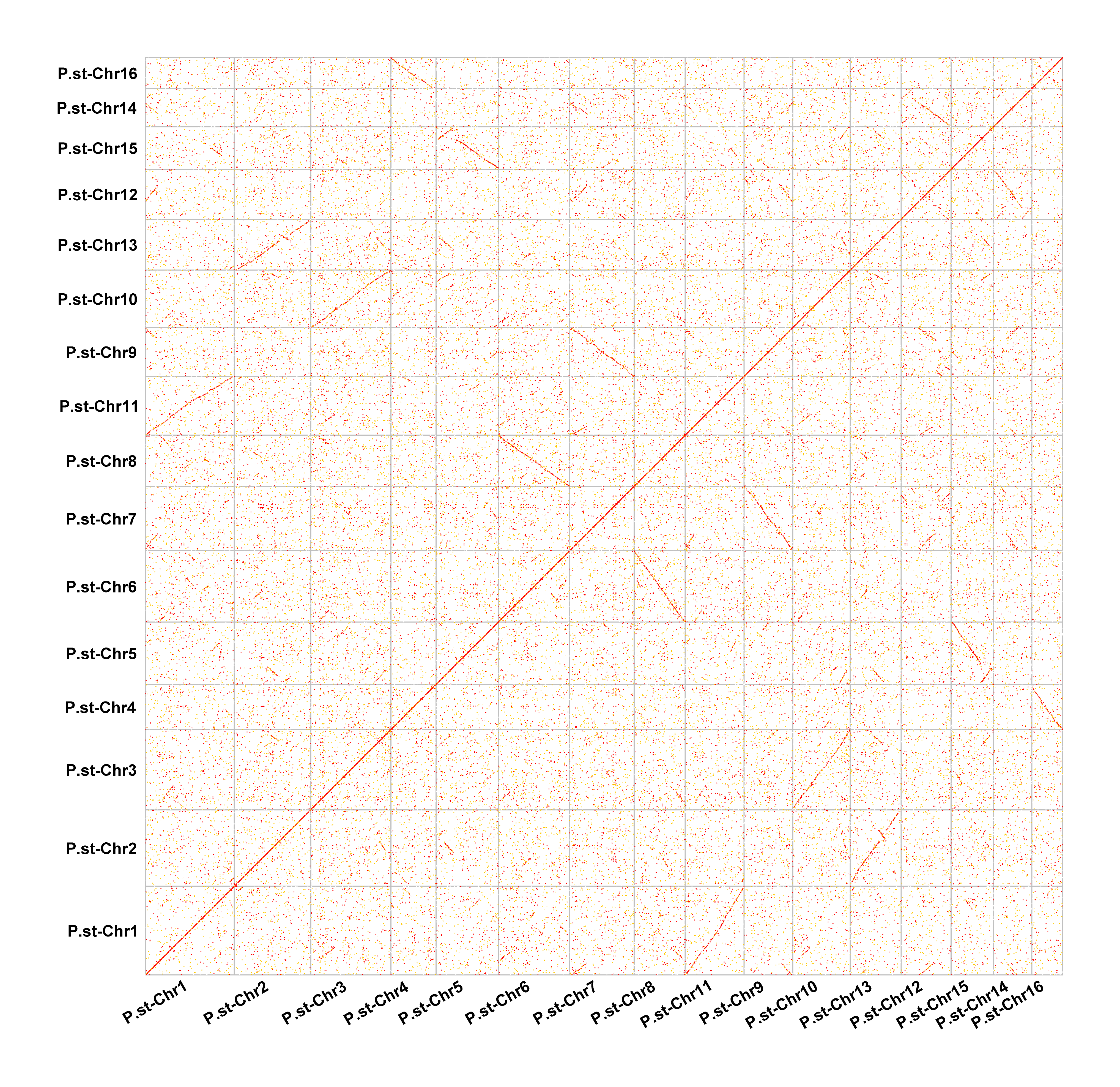

Supplement: qzae087_Supplementary_Data [file qzae087_supplementary_data.zip › Figure S6.tif]

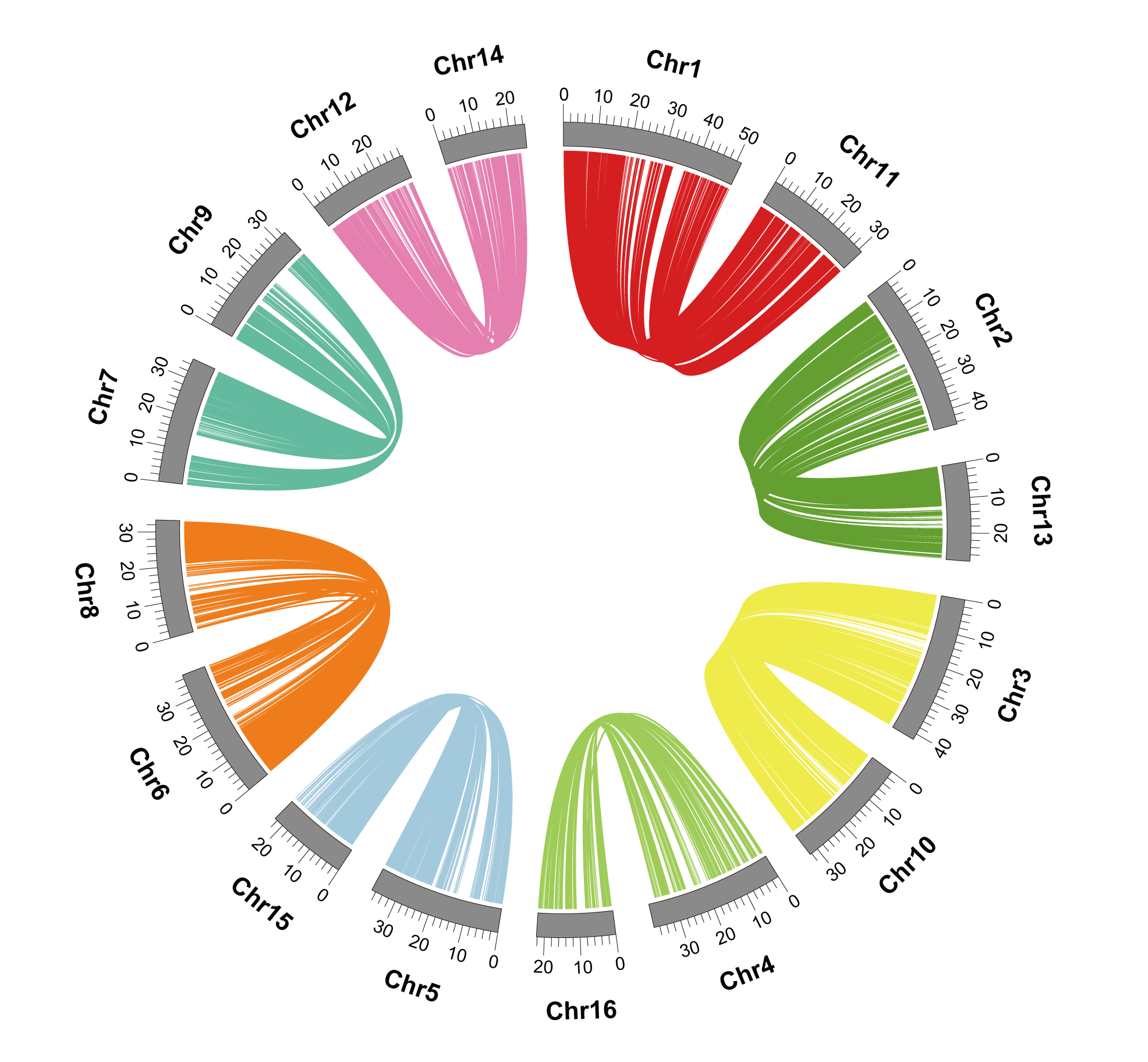

Supplement: qzae087_Supplementary_Data [file qzae087_supplementary_data.zip › Figure S7.tif]

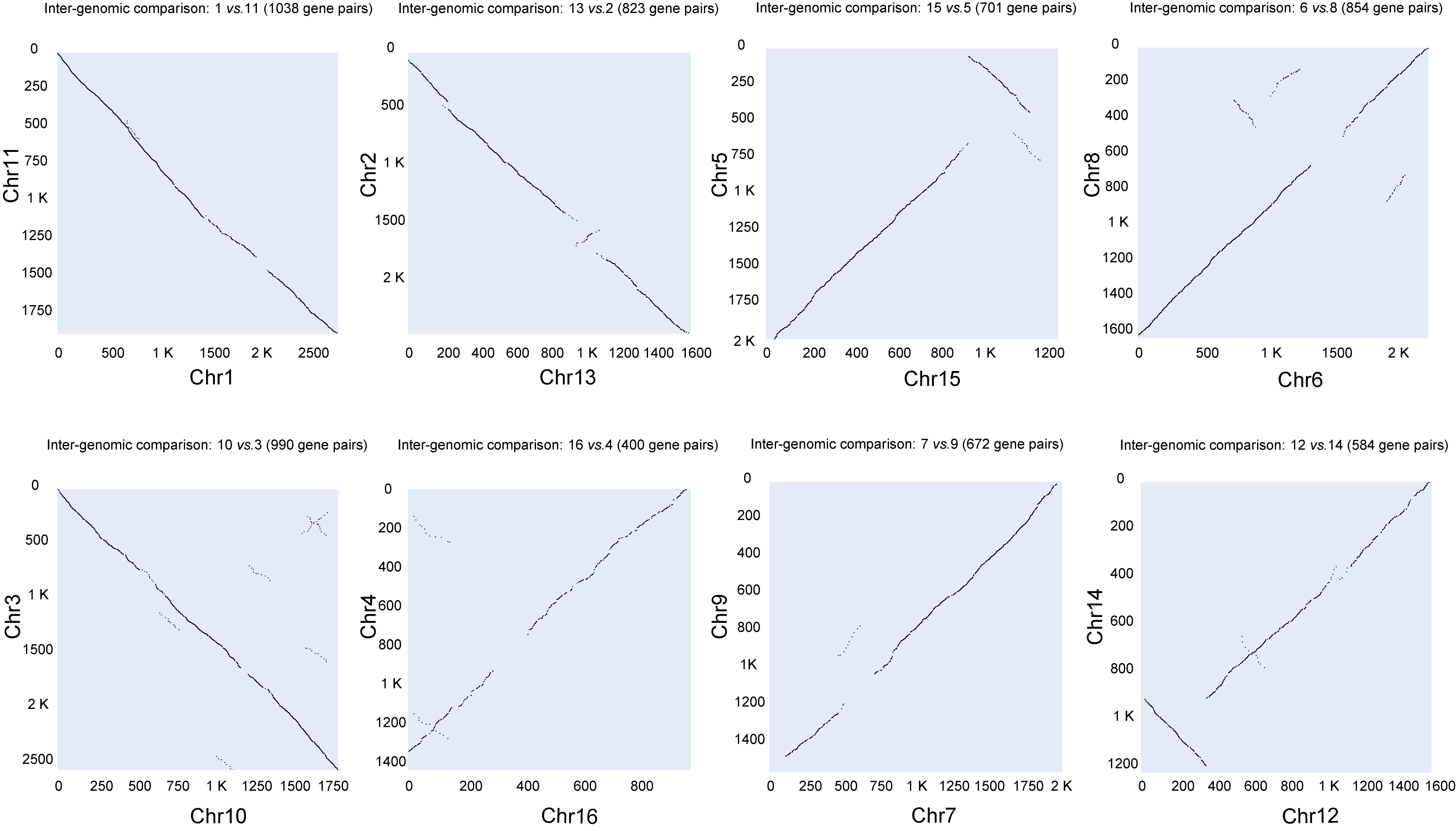

Supplement: qzae087_Supplementary_Data [file qzae087_supplementary_data.zip › Figure S8.tif]

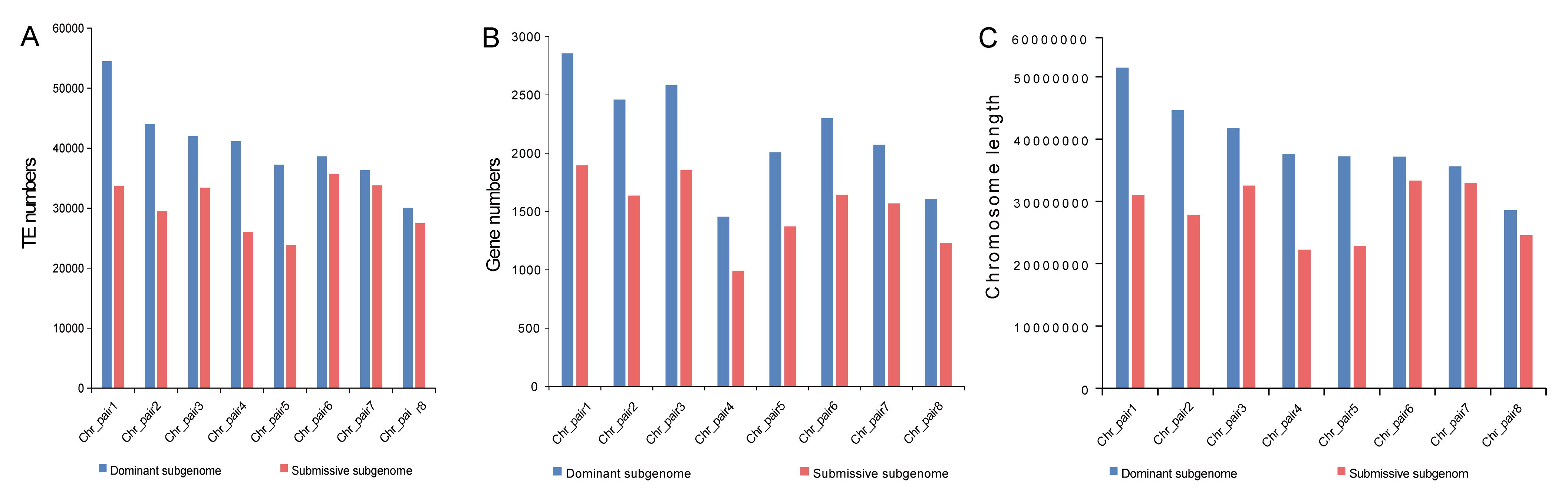

Supplement: qzae087_Supplementary_Data [file qzae087_supplementary_data.zip › Figure S9.tif]
